# A multigene phylogeny of *Olpidium *and its implications for early fungal evolution

**DOI:** 10.1186/1471-2148-11-331

**Published:** 2011-11-15

**Authors:** Satoshi Sekimoto, D'Ann Rochon, Jennifer E Long, Jaclyn M Dee, Mary L Berbee

**Affiliations:** 1 Department of Botany, 3529-6270 University Boulevard, University of British Columbia, Vancouver, British Columbia, V6T 1Z4 Canada; 2 Agriculture and Agri-Food Canada, Pacific Agri-Food Research Centre, Summerland, British Columbia, V0H 1Z0 Canada; 3 Department of Biological Sciences, The University of Alabama, Tuscaloosa, AL 35487, USA; 4 Department of Biology, University of Victoria, P.O. Box 3020 - Station CSC, Victoria, British Columbia, V8W 3N5 Canada

## Abstract

**Background:**

From a common ancestor with animals, the earliest fungi inherited flagellated zoospores for dispersal in water. Terrestrial fungi lost all flagellated stages and reproduce instead with nonmotile spores. *Olpidium virulentus *(= *Olpidium brassicae*), a unicellular fungus parasitizing vascular plant root cells, seemed anomalous. Although *Olpidium *produces zoospores, in previous phylogenetic studies it appeared nested among the terrestrial fungi. Its position was based mainly on ribosomal gene sequences and was not strongly supported. Our goal in this study was to use amino acid sequences from four genes to reconstruct the branching order of the early-diverging fungi with particular emphasis on the position of *Olpidium*.

**Results:**

We concatenated sequences from the *Ef-2*, *RPB1*, *RPB2 *and *actin *loci for maximum likelihood and Bayesian analyses. In the resulting trees, *Olpidium virulentus*, *O. bornovanus *and non-flagellated terrestrial fungi formed a strongly supported clade. Topology tests rejected monophyly of the *Olpidium *species with any other clades of flagellated fungi. Placing *Olpidium *at the base of terrestrial fungi was also rejected. Within the terrestrial fungi, *Olpidium *formed a monophyletic group with the taxa traditionally classified in the phylum Zygomycota. Within Zygomycota, Mucoromycotina was robustly monophyletic. Although without bootstrap support, Monoblepharidomycetes, a small class of zoosporic fungi, diverged from the basal node in Fungi. The zoosporic phylum Blastocladiomycota appeared as the sister group to the terrestrial fungi plus *Olpidium*.

**Conclusions:**

This study provides strong support for *Olpidium *as the closest living flagellated relative of the terrestrial fungi. Appearing nested among hyphal fungi, *Olpidium*'s unicellular thallus may have been derived from ancestral hyphae. Early in their evolution, terrestrial hyphal fungi may have reproduced with zoospores.

## Background

Fungi in modern ecosystems are able to cause plant diseases, serve as mycorrhizal partners to plants, or decompose litter and woody debris using the tubular hyphae (filaments of walled cells) that make up fungal bodies. Hyphae use hydrostatic pressure to penetrate tough substrates such as soil and plant tissue, secreting enzymes across their chitinous cell walls to break down complex organic compounds into simple, diffusible molecules that are absorbed to nourish growth. An increasing body of phylogenetic evidence indicates that fungi, animals, and protists, such as nucleariid amoebae and Ichthyosporea, all share a close common ancestor [1-3]. This pattern implies that the original fungus-like organisms were not terrestrial and hyphal in their assimilative phase but were instead aquatic, flagellated and unicellular.

Fungi that have been classified in Zygomycota are phylogenetically important because in most studies, they appear as the first terrestrial fungi to have evolved from flagellated, aquatic ancestors. However, their backbone relationships remain largely unresolved. The lack of decisive evidence for monophyly has led to alternative classifications for Zygomycota [4]. Fungi once placed in Zygomycota are sometimes distributed among the Glomeromycota, comprising the arbuscular mycorrhizal fungi [5-7]; the Mucoromycotina; and "Zygomycota, unresolved", which includes animal or fungal symbionts or pathogens in the subphyla Entomophthoromycotina, Zoopagomycotina, and Kickxellomycotina. For convenience in the text, we will continue to apply 'Zygomycota' to these terrestrial, non-flagellated fungi including Glomeromycota and Mucoromycotina.

In terms of evolutionary inference, some of the first molecular phylogenies from ribosomal gene sequences specified, probably incorrectly, that two clades of terrestrial Zygomycota evolved convergently from flagellated, aquatic ancestors. Early studies showed that the flagellated Blastocladiomycota grouped with terrestrial Zygomycota including *Rhizopus*, and the flagellated Chytridiomycota grouped with the terrestrial Zygomycota *Basidiobolus *[8-11]. This pattern was likely an artifact of long-branch attraction and it is contradicted by more recent analyses including more taxa or different loci. More recently, a phylogeny of the amino acid sequences of *RPB1 *showed *Basidiobolus *grouping with other Zygomycota rather than with Chytridiomycota [12]; Zygomycota appear monophyletic in analysis of *RPB1 *and *RPB2 *[13]; and Zygomycota are paraphyletic in a multi-locus, phylogenomic study [14].

Against the background of recent support for a single origin of nonflagellated terrestrial fungi (Zygomycota plus Dikarya), James et al. [5,15] found yet another possible example of convergent loss of flagella. As the first to include the zoospore-producing *Olpidium virulentus *(= *Olpidium brassicae*) [16] in their analyses, James et al. [5] were surprised to find that this flagellated fungus clustered with *Basidiobolus*, although without statistical support. *Olpidium *and *Basidiobolus *were further nested among terrestrial fungi with strong support from both posterior probabilities and likelihood bootstrap proportions. To explain the nesting of *Olpidium *within the non-flagellated fungi required 2-4 losses of flagella [5]. This finding of a flagellate within the terrestrial clade was no obvious artifact of long-branch attraction. The James et al. [5] study included a rich sampling of available basal fungal lineages and neither *Olpidium *nor *Basidiobolus *had particularly long-branch lengths.

**Figure 1:**
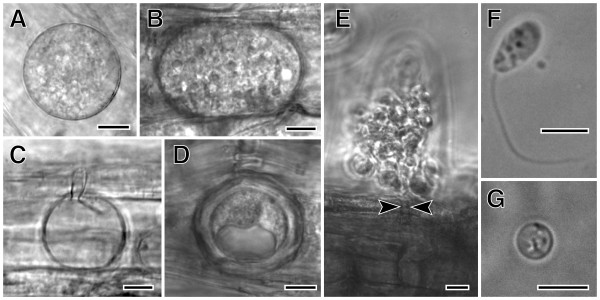
***Olpidium bornovanus*, a unicellular fungus, is an obligate parasite of plants that reproduces with flagellated, swimming zoospores**. A-B. Vegetative unicellular thalli in cucumber root cells. Thalli differentiate into sporangia with zoospores, or into resting spores. C. An empty sporangium, after zoospore release. D. A thick-walled resting spore. E. Zoospores being released from a sporangium, showing the sporangium exit tube (arrowheads). F. A swimming zoospore with a single posterior flagellum. G. An encysted zoospore. Bars: A-E = 10 μm; F,G = 5 μm.

*Olpidium *is however a challenging genus and it seemed possible that its apparent phylogenetic position was influenced by missing data. Several species, including *Olpidium virulentus *and *Olpidium bornovanus*, are biotrophic plant pathogens, unable to grow except as unicellular thalli that develop embedded inside living plant root cells [17-19]. At maturity, zoospores with single posterior flagella are liberated from the root cell through spore exit tubes [17] (Figure 1). Because they are biotrophic, relatively pure *Olpidium *DNA can only be harvested from zoospores. Washing roots with mature sporangia in distilled water triggers zoospore release. However, the zoospore suspension is not axenic. *Olpidium *DNA sequences are mostly too divergent to be amplified with universal fungal primers. As a result of these difficulties, James et al. [5] were only able to analyze one protein coding gene sequence (*RPB1*) in addition to ribosomal genes, from only one *Olpidium *species.

Our objectives in this study included rigorous testing of the phylogenetic position of *Olpidium *and resolving the relationships among clades in the Zygomycota and the flagellated fungi, with the overall goal of improving understanding of the early evolution of Fungi. We used genes for four proteins, translation elongation factor 2 (*Ef-2*), RNA polymerase II largest subunit (*RPB1*), RNA polymerase II second largest subunit (*RPB2*), and actin. Although *Ef-2 *genes have proven useful in other eukaryotic lineages [20], this study represents their first use for the deep phylogeny of Fungi. *Olpidium*, if nested within Zygomycota, becomes a key organism for reconstructing the trail of how terrestrial fungi lost their flagella and colonized land.

## Results

### Overall phylogenetic analyses of the kingdom Fungi

**Figure 2:**
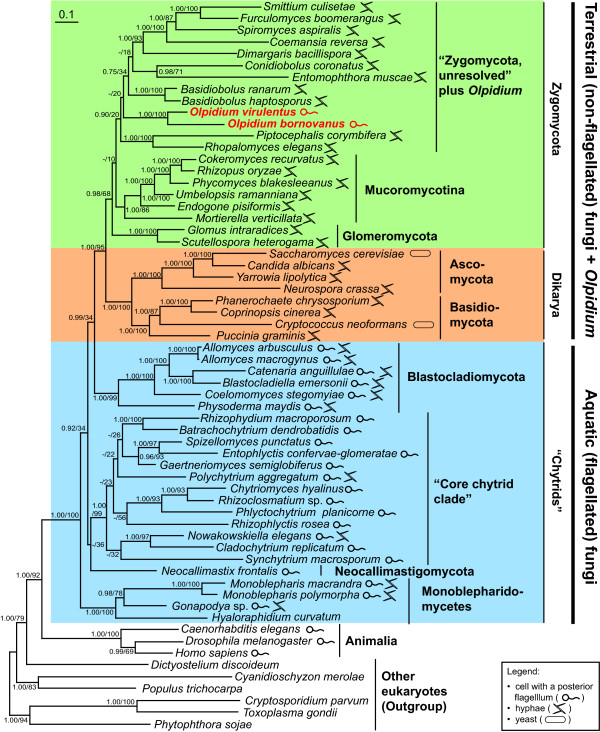
**A phylogeny from four protein-coding genes shows that *Olpidium *is the only flagellated genus in a clade of terrestrial non-flagellated fungi**. This maximum likelihood tree from RAxML is based on concatenated amino acid sequences of genes for the elongation factor 2, the RNA polymerase II largest and second largest subunits, and actin. Numbers on the internal nodes represent posterior probabilities and bootstrap percentages calculated by MrBayes and RAxML, respectively.

The kingdom Fungi formed a robust clade in maximum likelihood and Bayesian analysis from the dataset of concatenated amino acid sequences from four genes (Figure 2). The terrestrial fungi plus the flagellated fungi *Olpidium virulentus *and *O. bornovanus *formed a monophyletic group excluding all other flagellated fungi with 95% bootstrap support and a posterior probability of 1.0 (Figure 2). Topology tests rejected all alternative trees that constrained the *Olpidium *species to cluster with other groups of flagellated fungi (Table 1). A clade including the Zygomycota plus *Olpidium *was also monophyletic with 68% bootstrap support from likelihood and a posterior probability of 0.98 (Figure 2). In our analysis, terrestrial fungi were divided between the monophyletic Zygomycota plus *Olpidium*, and the Dikarya (= Ascomycota plus Basidiomycota). The Zygomycota included two well-supported groups: Mucoromycotina and Glomeromycota. The Zygomycota also included "Zygomycota, unresolved" (Figure 2), a weakly supported clade consisting largely of animal or fungal symbionts or pathogens [4-6]. "Zygomycota, unresolved" also included *Olpidium*. If any group within Zygomycota were the sister group to *Olpidium*, it was not clear from our phylogenies. The two species that clustered most closely with *Olpidium *in the Bayesian analysis, *Piptocephalis corymbifera *and *Rhopalomyces elegans*, had long-branches (data not shown), were missing data from two loci, and did not cluster with *Olpidium *in the likelihood analysis (Figure 2). In Approximately Unbiased tree topology tests, uniting *Olpidium *with Glomeromycota could not be rejected at the p-value of < 0.05 (Table 1). In the more conservative weighted Shimodaira-Hasegawa tests, uniting *Olpidium *with Mucoromycotina and with Dikarya could not be rejected either (Table 1). These analyses suggest that *Olpidium *is part of the terrestrial fungi, but leave open the possibility that it may be the sister taxon to the Dikarya, or sister to one of the basal clades within Zygomycota.

**Table 1:** The only phylogenetic positions of *Olpidium *that could not be rejected by either the weighted Shimodaira-Hasegawa (wSH) or the Approximately Unbiased (AU) tests were within the Zygomycota.

Tree	Constraint	ΔlnL	AU^1^	wSH^1^
1	*Olpidium *united with "Zygomycota, unresolved" (Best tree; Figure 2)	0.0	0.896	0.995
2	*Olpidium *united with Glomeromycota in Zygomycota	19.8	0.208	0.619
3	*Olpidium *united with Mucoromycotina in Zygomycota	42.3	0.013*	0.087
4	*Olpidium *united with Dikarya	65.7	0.011*	0.058
5	*Olpidium *sister to all terrestrial fungi	80.8	0.000**	0.010*
6	*Olpidium *united with Blastocladiomycota	148.4	0.002**	0.004**
7	*Olpidium *sister to all other fungi	148.7	0.005**	0.008**
8	*Olpidium *sister to all terrestrial fungi and Blastocladiomycota	154.6	0.000**	0.000**
9	*Olpidium *united with Neocallimastigomycota	174.1	0.004**	0.013*
10	*Olpidium *united with Monoblepharidomycetes	203.6	0.000**	0.000**
11	*Olpidium *united with "Core chytrid clade"	233.6	0.000**	0.000**

^1^AU, Approximately Unbiased; wSH, weighted Shimodaira-Hasegawa test. The constrained tree was significantly worse than the best tree (Figure 2) at P < 0.05* or P < 0.01**.

Concerning the other flagellated fungi, the concatenated dataset provided strong support for the monophyly of the Blastocladiomycota, the "core chytrid clade", and the Monoblepharidomycetes. Although support was low, the flagellated fungi form a paraphyletic assemblage, with Monoblepharidomycetes diverging first, then the Neocallimastigomycota, the "core chytrid clade," and finally, the Blastocladiomycota as the sister group to the terrestrial fungi. Tree topology tests did not reject any of the alternative possible patterns of relationships among the three basal clades of flagellated fungi (Monoblepharidomycetes, Neocallimastigomycota, and "core chytrid clade") (data not shown).

Analyzed separately, individual gene trees did not resolve branching order of basal fungal clades with significant bootstrap support (Figure S1a-S1d in Additional file 1). However, the Ef-2 likelihood tree showed *Olpidium *in "Zygomycota, unresolved" with 56% support and all the genes except actin placed *Olpidium *with members of "Zygomycota, unresolved". Overall, actin provided less phylogenetic information than the other genes due to its low substitution rates (note the much longer scale bar relative to 0.1 substitutions, Figure S1d) compared with scale bars for other single genes (Figure S1a-S1c) for phylogenetic analysis.

### *Olpidium *sequences

Because *Olpidium *cultures were not axenic, we had to use phylogenetic concordance as a criterion for testing whether or not their DNA sequences could be from contaminants. Reassuringly, in a maximum likelihood tree from concatenated data (Figure 2), sequences of the two *Olpidium *species formed a robust clade among other fungi with 100% bootstrap support. As expected, they grouped among the Zygomycota, consistent with results from James et al. [5]. Sequences from both species also clustered together in individual gene trees whenever both species were included in the analysis (Figure S1a, S1b, and S1d in Additional file 1). The *RPB1 *gene sequence obtained from our *O.virulentus *(GenBank AB644405) was almost identical to *O.virulentus *isolate used in James et al. (GenBank DQ294609) [5]. As an additional assay for possible contamination, we amplified the SSU rRNA gene region from DNA extracts by PCR with eukaryotic-universal SSU primers [SR1 (5'-TACCTGGTTGATCCTGCCAG-3') and SR12 (5'-CCTTCCGCAGGTTCACCTAC-3')], then sequenced amplicons directly. We found the *Olpidium *SSU rRNA gene region in our extracts and we did not detect the host plant or any other sequences (data not shown). This result suggested that *Olpidium *DNA predominated in our extracts.

However, along with the *Olpidium *sequences that clustered, as expected, in the fungi, we also found additional, aberrant sequences for *Ef-2*, *RPB2*, and *actin *(Figure S1a, S1c, and S1d in Additional file 1). These sequences did not match common laboratory contaminants. From the *Ef-2 *dataset, in addition to the set of *Olpidium *sequences that clustered as expected, within "Zygomycota, unresolved," a pair of sequences clustered with the slime mold *Dictyostelium discoideum *(Figure S1a). From the *RPB2 *dataset, one *O. bornovanus *sequence clustered with the zygomycete *Conidiobolus coronatus *and the other with *D. discoideum *(Figure S1c). In the actin dataset, we detected two *O. virulentus *and four *O. bornovanus *actin-like sequences (Figure S1d). One pair of sequences from *Olpidium *species clustered together in Fungi, but the other four sequences clustered with non-fungal taxa (Figure S1d). The aberrant sequences could be divergent paralogs or genes gained through horizontal gene transfer, and we cannot even rule out contamination as their source. We deleted those aberrant sequences (Figure S1a, S1c, S1d) from our four-protein dataset used in the likelihood and Bayesian analyses (Figure 2, S2).

## Discussion

### Phylogenetic position of *Olpidium*

Our likelihood and Bayesian analyses strongly suggest that *Olpidium virulentus *and *O. bornovanus *are more closely related to terrestrial fungi than to other clades of flagellated fungi. *Olpidium *has typical "core chytrid" characters including a single endobiotic sporangium producing zoospores having a single posterior flagellum. It shares no obvious morphological characters with any clade of terrestrial fungi. Whether *Olpidium *and terrestrial fungi share biochemical characters, such as enzyme systems for entry into plant cell walls, remains to be seen.

None of the other Zygomycota genera are close to *Olpidium*, based on the tendency of *Olpidium *to cluster with different genera in different analyses, without support. This lack of resolution may be yet another long-branch attraction problem. Adding more species of *Olpidium *to a phylogeny would contribute to breaking up the *Olpidium *branch and would allow reconstruction of the ancestral traits of *Olpidium*. However, most of the ~50 *Olpidium *species are difficult to obtain. We were able to include *O. virulentus *and *O. bornovanus *in this study only because Rochon and colleagues grow them routinely to serve as vectors for experimental transmission of viral diseases of plants [21,22]. Other species of evolutionary interest from other hosts (fungi, moss protonemata, microscopic animals, or algae) would have to be isolated from nature and cultured along with their hosts. As shown in other morphologically simple but diverse fungal genera, e.g., *Rhizophydium *[23], *Olpidium *is likely not monophyletic. Species differ in cell size (depending on the size of the host cell), shape (spherical to ovoid, irregular, tubular or elongate), and other morphological characters (resting spore morphology, number of spore exit tubes etc.). Ultrastructure also varies from species to species. *Olpidium pendulum *perhaps belongs in a genus separate from *O. virulentus *and *O. bornovanus *(as a synonym of *O. cucurbitacearum*) [24-31]. Some of the other species might belong in the same clade as our two plant parasitic *Olpidium*, while others will likely fall out with the "core chytrids" instead.

### Terrestrial fungal relationships--a classical phylogenetic challenge?

Conflicts among phylogenies from different datasets suggest that as fungi first colonized land, at least five basal lineages (Mucoromycotina, Glomeromycota, "Zygomycota, unresolved", Dikarya, *Olpidium*) radiated rapidly and then evolved independently by different rates and modes of substitution. A succession of researchers have noted and attempted to solve the problem of variation in rates and modes with various analytical strategies. Tanabe et al. [32] pointed out the ribosomal substitution rates varied dramatically in relative rate tests and recommended relying more heavily on *RPB1*, which showed less rate variation. Voigt and Wöstemeyer [33] used logdet methods to overcome biases resulting from lineage-specific variation in nucleotide composition to estimate distances. Liu et al. [14] applied huge amounts of sequence, analyzing 40,925 amino acids with a substitution model intended to minimize long-branch attraction problems. In their ribosomal gene phylogeny, White et al. [6] included an excellent sampling of taxa representing most known Zygomycota lineages, including many species that have not appeared in other studies because they are difficult to grow.

No aspect of basal branching order for these taxa receives consistent support across studies. The clade corresponding to the traditional Zygomycota from our study and from Liu et al.'s *RPB1 *and *RPB2 *amino acid phylogenies [13] does not appear in analyses consisting largely or entirely of ribosomal DNA sequences [5,6,15] or of *actin *plus *Ef-1 alpha *[33,34]. It is also missing from a phylogenomic study of nuclear genes [14] where Mucoromycotina is sister to Dikarya. Our analyses show *Mortierella verticillata *as part of Mucoromycotina. This relationship was supported strongly in our analysis of concatenated data; it was evident in our *Ef-2 *and *RPB1 *gene trees, and it is consistent with some traditional classifications schemes based on morphology [35]. Liu et al. [14], however, showed *Mortierella *diverging much earlier (although with limited support) as one of the three Zygomycota lineages paraphyletic to Mucoromycotina plus Dikarya. Although the Liu et al. [14] study had a great deal of data per taxon and an appropriate model of evolution, the number of genomes available for analysis may still be too small to capture branching order with statistical support. As a result, in Liu et al.'s [14] study, long-branch attraction or other kinds of systematic error may have been responsible for pulling *Mortierella *away from its closest relatives. In contrast, our phylogenies have good representation of lineages but data from only four loci, and our inability to reject alternative topologies in weighted Shimodaira-Hasegawa and Approximately Unbiased tests reflects the need for more data per taxon. Resolution of the relationships among early fungi joins the resolution of relationships among the first animals and among seed plants as a difficult phylogenetic problem.

### Phylogenetic age of origin of fungal hyphae

Branching order among early-diverging fungal groups has been assumed to involve progressive elaboration of thread-like hyphal systems [2], from ancestors, which like most "core chytrids," had unicellular thalli. Beyond the Fungi, the oomycetes, chromistan fungus-like protists, provide an example of convergent origin of hyphae. Oomycete thalli range from single cells to well-defined mycelia [19]. Recent phylogenetic studies have indicated that the ancestral oomycetes might have been unicellular endoparasites of marine organisms that later gave rise to hyphal species. Of the hyphal oomycete species, some remained aquatic, while others invaded land [36]. Ability to form hyphae may have been an important character that allowed early oomycetes as well as early Fungi to poke, penetrate and explore to find terrestrial food that may have been patchy in its distribution.

As in the oomycetes, hyphae may have evolved in fungi even before they colonized land. Most of the known Monoblepharidomycetes are hyphal, and they diverged at or near the base of the Fungi. The Blastocladiomycota, the aquatic sister clade to the terrestrial fungi [5,6,14], also includes genera with well-developed mycelia. Since its relatives are hyphal, *Olpidium*'s unicellular thallus is perhaps an adaptation to parasitism and a reduction from a hyphal ancestor. On the other hand, finding *Olpidium *nested among the terrestrial clades implies that fungi on land initially had flagellated spores. Eukaryotic flagella are complex structures that would have been unlikely to evolve repeatedly, and so multiple losses of flagella were far more likely than a convergent gain in *Olpidium*. As in early terrestrial animals and plants, early terrestrial fungi retained a motile unicellular phase.

We reluctantly excluded two interesting basal fungal lineages, microsporidia and *Rozella *[5], from our analyses. Microsporidia, obligate intracellular pathogens of animals, have unusual, highly divergent genes [37]. Conserved synteny among the microsporidia and the zygomycotan *Phycomyces blakesleeanus *and *Rhizopus oryzae *has been used to suggest that the microsporidia may be related to Mucoromycotina [38], an interpretation contested by Koestler and Ebersberger [39]. Sequences from microsporidia are difficult to align and a source of artifacts involving long-branch attraction. It seemed more likely that microsporidia would obscure phylogenetic signal in our dataset than that our analysis would correctly resolve their relationships.

*Rozella allomycis *is a unicellular obligate endoparasite of *Allomyces arbusculus*, another fungus [40]. James et al. [5] found *Rozella allomycis *to group with microsporidia at the base of Fungi. Unfortunately, the only known living culture of *Rozella *had died before our study, so we were unable to contribute new data for the genus. *Rozella *is basal in Fungi in our *RPB1 *and *RPB2 *individual trees. This could be its correct phylogenetic position but it could also reflect long-branch attraction (Figure S1b, S1c, in Additional file 1). Adding *Rozella *to the analysis caused relationships among the other flagellated fungi to shift (Figure S2 in Additional file 2). Our tree topology test did not rule out most possible topologies for the positioning of *Rozella *with other fungal lineages (Table S1, in Additional file 2). Uncertain about the reason for the behaviour of *Rozella*, we show the consequences of including it in the supplemental file (Figure S2) but excluded the taxon from the analyses in Figure 2.

## Conclusions

Even with analysis of amino acid sequences from four protein-coding loci, and from all available early-diverging lineages, the resolution of branching order in the deepest parts of kingdom Fungi remains uncertain. Instead of converging on a single solution, recent studies of early fungi have produced conflicting phylogenies. Our trees showed the zoosporic Monoblepharidomycetes to be sister to all other fungi, the zoosporic Blastocladiomycota to be sister to terrestrial fungi, and the Zygomycota to be monophyletic. These relationships require further testing through phylogenomic analysis and comprehensive taxon sampling.

Our study provided the strongest support to date for the monophyletic group that includes two zoosporic *Olpidium *species together with all of the non-flagellated, terrestrial fungi. Here, the results from our study and others are congruent. If its nested position among hyphal fungi is correct, the unicellular thallus of *Olpidium *may have evolved by reduction from hyphae, contradicting an intuitive expectation that the smaller structure would predate the larger one. Swimming zoospores that germinated and grew as hyphae may have been the fungal analogues of amphibians that made the earliest evolutionary forays into drier environments.

## Methods

### Fungal strains, DNA and RNA extraction, PCR amplification and sequencing

Table S2 (in Additional file 3) lists fungal strains sequenced in this study. All zygomycetes were maintained on potato dextrose agar medium (PDA: Difco Grade, Becton, Dickinson and Company, MD, USA), or PDA plus 0.5% yeast extract medium at ambient temperature, as described in O'Donnell [41]. All chytrids except the two *Olpidium *strains were maintained on PmTG agar medium [42]: 1g peptonized milk, 1g tryptone, 5g glucose, 8g agar, and 1litre of distilled water) at ambient temperature. *Olpidium bornovanus *was maintained on cucumber roots (*C. sativis *cv. Poinsette 76), and *O. virulentus *was maintained on lettuce roots (*Lactuca sativa *cv. White Boston) as described by Campbell et al. [43]. The full length of the internal transcribed spacer (ITS) regions (between the *18S*, *5.8S *and *28S ribosomal RNA *genes) of our *O. virulentus *strain was sequenced for species identification, and it had 100% nucleotide similarity with that of *O. virulentus *strain GBR1 (GenBank no. AY373011) [16,44]. *Ef-2 *gene sequences of two *Olpidium *species and *RPB2 *gene sequences of *O. bornovanus *were obtained from total RNA, and all other sequences obtained in this study were from total genomic DNA. For the RNA and DNA extraction from two *Olpidium *strains, we used TRIzol Reagent following the procedure outlined by Invitrogen (Mississauga, Ontario, Canada). Prior to DNA and RNA extraction the *Olpidium *zoospores were pelleted from root washings at 2,700 × g for 7 minutes. Total genomic DNA from cultured strains was extracted using a DNeasy Plant Mini Kit (Qiagen, Mississauga, Ontario, Canada), following the manufacturer's protocol.

Primers were designed in this study, or taken from James et al. [5], or Hoffmann et al. [45] (Table S2, Additional file 3 Table S3, Additional file 4). We amplified the partial genes for *eukaryotic translation elongation factor 2 *(*Ef-2*), *RNA polymerase II largest subunit *(*RPB1*), *RNA polymerase II second largest subunit *(*RPB2*), and *actin *using 0.5 μM concentrations of primers, two to five μl of genomic DNA solution, and PureTaq™ Ready-To-Go™ PCR beads (Amersham Biosciences, Piscataway, NJ, USA) following the manufacturer's protocol. Total PCR reaction volume was 25 μl, and cycling parameters were: initial denaturation (5 min, 94°C), followed by 40 cycles (94°C, 10 s; 50-65°C, 20 s; 72°C for 30 s plus 4 additional seconds per cycle), and then a final extension at 72°C for 7 min. RT-PCR was conducted using SuperScript™ One-Step RT-PCR with Platinum^® ^*Taq *(Invitrogen) following manufacturer's protocol. The total 25 μl reaction volume of RT-PCR, contained 0.2 μM concentrations of primers, 1 μl of total RNA solution, 0.5 μl of RNaseOUT^® ^Recombinant Ribonuclease Inhibitor (Invitrogen), 12.5 μl of 2× Reaction Mix, and 1.0 μl of RT/Platinum^® ^*Taq *Mix. Cycling parameters were: reverse transcription (32 min, 55°C) and initial denaturation (5 min, 94°C), followed by 40 cycles (94°C, 15 s; 55°C, 30 s; 68°C for 30 s plus 4 additional seconds per cycle), and then a final extension at 68°C for 10 min. PCR products were then purified with EtOH precipitation (20 μl PCR products, 2 μl 3 M sodium acetate (pH 4.5), 50 μl 95% ethanol; 15 min spin and rinse with 70% ethanol twice, resuspended in 20 μl water), or cloned with TOPO^® ^TA Cloning kit (Invitrogen) following the manufacturer's protocol. Products were then sequenced with BigDye Terminator v3.1 Cycle Sequencing Kit on a Applied Biosystems 3730S 48-capillary sequencer (Applied Biosystems, Foster City, CA, USA) at NAPS Unit, MSL, University of British Columbia [accession numbers; DDBJ:AB609150 - AB609186, AB625456, and GenBank:HM117701 - HM117719; Table S4 in Additional file 5].

### Sequence alignments

DNA sequences were assembled and edited using the software Se-Al v2.0a11 [46]. Also using Se-Al, sequences were manually added to *RPB1 *and *RPB2 *amino acid alignments of James et al. [5] or to the actin alignment of Voigt & Wöstemeyer [33]. Introns, ambiguously aligned positions and gaps were excluded from both analyses. Alignments have been accessioned in TreeBASE (S11208, http://purl.org/phylo/treebase/phylows/study/TB2:S11208).

### Molecular phylogenetic analysis

Phylogenetic relationships were inferred from maximum likelihood and Bayesian methods, and all four protein datasets were combined and used for both analyses (Figure 2). ProtTest version 2.4 [47] estimated that the best-fit model of protein evolution for each of the individual alignment datasets was the LG model, with site-to-site rate variation approximated with a gamma distribution (G) and an estimated proportion of invariable sites (I), and with empirical base frequencies (F) (LG+G+I+F). For likelihood, we used RAxML version 7.0.3 [48], with the LG+G+I+F model and 600 replicate searches. We used 1000 likelihood bootstrap replicates with the rapid bootstrapping algorithm in RAxML version 7.2.7 with the LG+G+F model conducted on CIPRES Science Gateway Web server (on RAxML-HPC2 on Abe 7.2.7) [49]. For Bayesian posterior probabilities for branch nodes, we used MrBayes version 3.1.2 [50] on Parallel MrBayes Web Server at the BioHPC compute cluster at CBSU (http://biohpc.org/), with 1,713,600 generations, sampling trees every 100 generations, and discarding the first 5000 trees as a burnin. Convergence was evaluated from running two independent chains. The effective sample size was > 288.3 and 325.2, estimated by Tracer v1.5 [51]. Each of the four of individual protein data sets was analyzed with likelihood as explained above Figure S1a-S1d (in Additional file 1).

### Tree topology tests

To compare alternative phylogenetic positions for *Olpidium*, we used the Approximately Unbiased test and the weighted Shimodaira-Hasegawa test [52,53], both implemented by CONSEL v0.20 [54], using the site-wise likelihood values estimated in PAUP v.4.0b10 [55]. Each constrained tree was based on an initial guide tree with a single internal branch, generated in MacClade version 4.08 [56] or in Mesquite version 2.75 [57] (Additional file 6). The most likely tree (Additional file 6), given each constraint, was found using 50 search replicates in RAxML version 7.2.8 with the LG+G+F model conducted on CIPRES Science Gateway Web server (on RAxML-HPC2 on TeraGrid) [49].

## Authors' contributions

SS and JMD sequenced *Ef-2 *gene sequences. SS sequenced *RPB1 *and *RPB2 *gene sequences. JEL sequenced and constructed actin data set. SS constructed *Ef-2*, *RPB1*, and *RPB2 *dataset, and prepared the manuscript. DR prepared and provided DNA and RNA extracts from two *Olpidium *species. SS and MLB conducted the phylogenetic analysis. MLB participated in the design of study. All authors joined in reading, editing and approving the manuscript.

## Supplementary Material

Additional file 1 **Figure S1. The phylogeny of the kingdom Fungi based on likelihood analysis of amino acid sequences from single genes. Figure S1a, elongation factor 2 tree; Figure S1b, RNA polymerase II largest subunit tree; Figure S1c, RNA polymerase II second largest subunit tree; Figure S1d, actin tree**. Click here for file

Additional file 2 **Figure S2 and Table S1. Figure S2. The phylogeny of the kingdom Fungi, including *Rozella allomycis*, based on likelihood analysis of amino acid sequences of four concatenated protein-encoding genes. Table S1. Tree topology tests showing that most of the alternative phylogenetic positions of *Rozella *could not be rejected**. Click here for file

Additional file 3 **Table S2. Species sequenced in this study, including voucher numbers and primer sets used**. Click here for file

Additional file 4 **Table S3. Primers used in this study**. Click here for file

Additional file 5 **Table S4. GenBank accession numbers of sequences used in this study**. Click here for file

Additional file 6 **The initial guide trees with a single internal branch, and the most likely trees given the constraint, that we used for the tree topology tests in Table **1**. All trees are in Newick format**. Click here for file
