# Supplementary material for: A multigene phylogeny of Olpidium and its implications for early fungal evolution
Source: BMC Evol Biol. 2011 Nov 15;11:331. doi: 10.1186/1471-2148-11-331 (PMC3247622; doi:10.1186/1471-2148-11-331)
Supplement: Additional file 1 — Figure S1. The phylogeny of the kingdom Fungi based on likelihood analysis of amino acid sequences from single genes. Figure S1a, elongation factor 2 tree; Figure S1b, RNA polymerase II largest subunit tree; Figure S1c, RNA polymerase II second largest subunit tree; Figure S1d, actin tree. [file 1471-2148-11-331-S1.PDF]

Sekimoto *et al.* A multigene phylogeny of *Olpidium* and its implications for early fungal evolution.

Additional file 1

Figure S1. The phylogeny of the kingdom Fungi based on individual protein datasets. Maximum likelihood trees were from thorough likelihood searches ("-f a" option) using the model that fitted best, LG+G+F. Numbers at the branches represent support above 50% from 1000 rapid bootstrap replicates. Searches were conducted on CIPRES Science Gateway Web server (with RAxML-HPC2 on Abe 7.2.7; Stamatakis et al. [49]). Two *Olpidium* species are indicated in *bold red*.

Figure S1a. The ML tree based on eukaryotic translation elongation factor 2 (Ef-2) protein dataset (48 OTUs, 823 sites).

Figure S1b. The ML tree based on RNA polymerase II largest subunit (RPB1) protein dataset (59 OTUs, 977 sites).

Figure S1c. The ML tree based on RNA polymerase II second largest subunit (RPB2) protein dataset (58 OTUs, 886 sites).

Figure S1d. The ML tree based on actin protein dataset (47 OTUs, 287 sites).

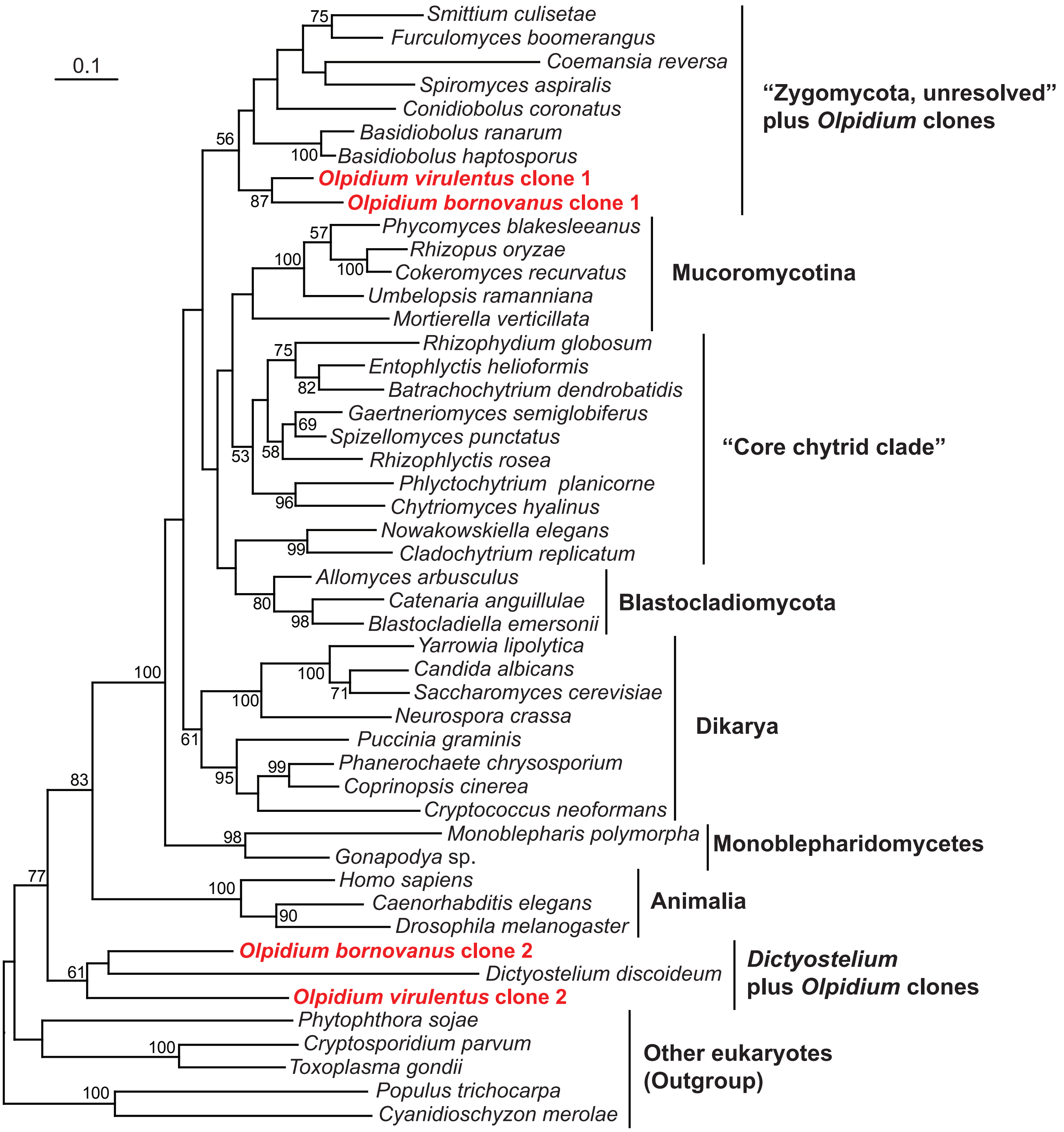
0.1
Smittium culisetae
Furculomyces boomerangus
Coemansia reversa
Spiromyces aspiralis
Conidiobolus coronatus
Basidiobolus ranarum
Basidiobolus haptosporus
Olpidium virulentus clone 1
Olpidium bornovanus clone 1
"Zygomycota, unresolved" plus Olpidium clones
Phycomyces blakesleeanus
Rhizopus oryzae
Cokeromyces recurvatus
Umbelopsis ramanniana
Mortierella verticillata
Mucoromycotina
Rhizophydium globosum
Entophlyctis helioformis
Batrachochytrium dendrobatidis
Gaertneriomyces semiglobiferus
Spizellomyces punctatus
Rhizophlyctis rosea
Phlyctochytrium planicorne
Chytriomyces hyalinus
Nowakowskiella elegans
Cladochytrium replicatum
"Core chytrid clade"
Allomyces arbusculus
Catenaria anguillulae
Blastocladiella emersonii
Blastocladiomycota
Yarrowia lipolytica
Candida albicans
Saccharomyces cerevisiae
Neurospora crassa
Puccinia graminis
Phanerochaete chrysosporium
Coprinopsis cinerea
Cryptococcus neoformans
Dikarya
Monoblepharis polymorpha
Gonapodya sp.
Monoblepharidomycetes
Homo sapiens
Caenorhabditis elegans
Drosophila melanogaster
Animalia
Olpidium bornovanus clone 2
Dictyostelium discoideum
Olpidium virulentus clone 2
Dictyostelium plus Olpidium clones
Phytophthora sojae
Cryptosporidium parvum
Toxoplasma gondii
Populus trichocarpa
Cyanidioschyzon merolae
Other eukaryotes (Outgroup)
75
100
56
87
57
100
100
75
82
69
58
53
96
99
80
98
100
71
100
61
99
95
100
98
83
100
90
77
61
100
100

Figure S1a

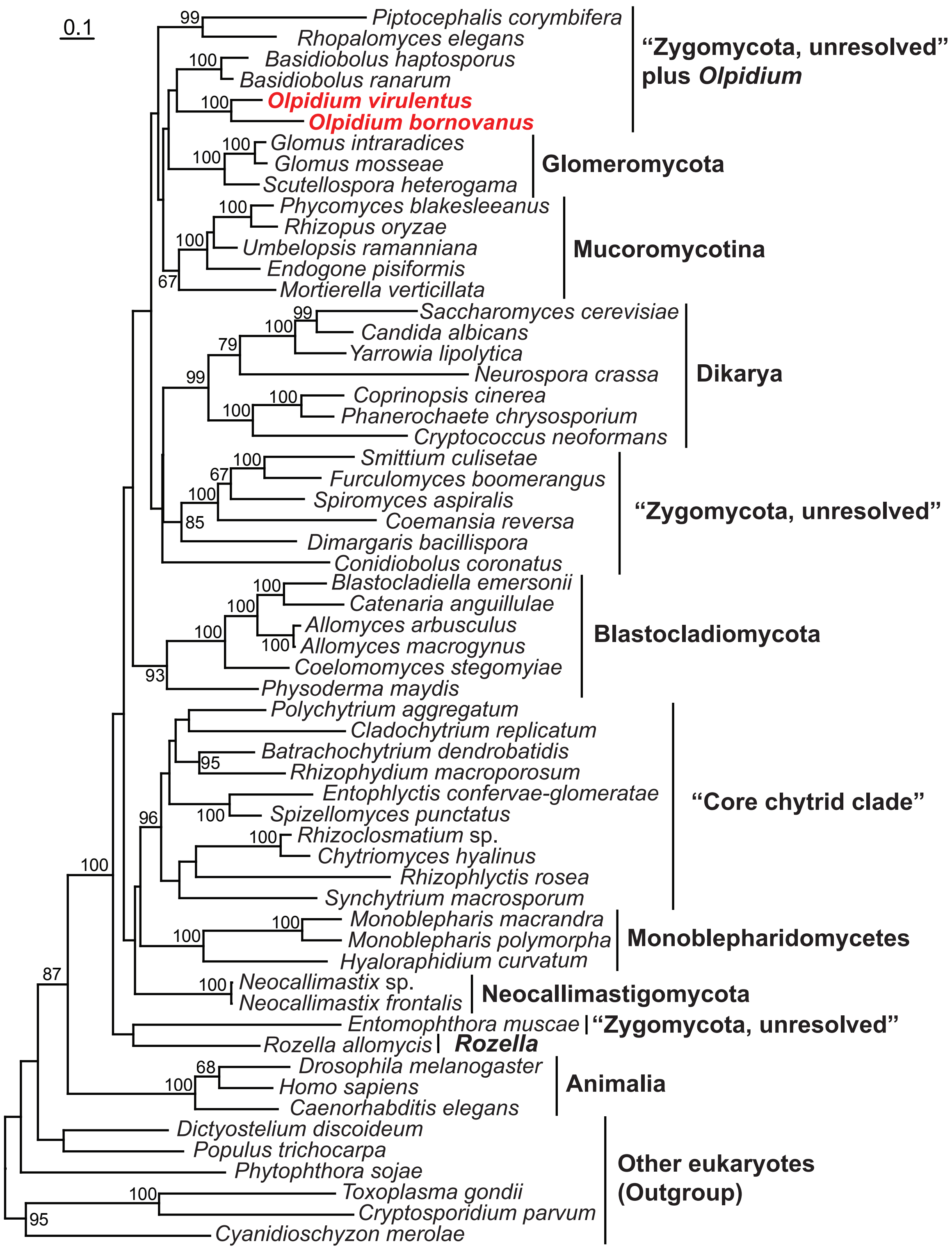
0.1
Piptocephalis corymbifera
Rhopalomyces elegans
Basidiobolus haptosporus
Basidiobolus ranarum
Olpidium virulentus
Olpidium bornovanus
"Zygomycota, unresolved" plus Olpidium
Glomus intraradices
Glomus mosseae
Scutellospora heterogama
Glomeromycota
Phycomyces blakesleeanus
Rhizopus oryzae
Umbelopsis ramanniana
Endogone pisiformis
Mortierella verticillata
Mucoromycotina
Saccharomyces cerevisiae
Candida albicans
Yarrowia lipolytica
Neurospora crassa
Coprinopsis cinerea
Phanerochaete chrysosporium
Cryptococcus neoformans
Dikarya
Smittium culisetae
Furculomyces boomerangus
Spiromyces aspiralis
Coemansia reversa
Dimargaris bacillispora
Conidiobolus coronatus
"Zygomycota, unresolved"
Blastocladiella emersonii
Catenaria anguillulae
Allomyces arbusculus
Allomyces macrogynus
Coelomomyces stegomyiae
Physoderma maydis
Blastocladiomycota
Polychytrium aggregatum
Cladochytrium replicatum
Batrachochytrium dendrobatidis
Rhizophydium macroporosum
Entophlyctis confervae-glomeratae
Spizellomyces punctatus
Rhizoclosmatium sp.
Chytriomyces hyalinus
Rhizophlyctis rosea
Synchytrium macrosporum
"Core chytrid clade"
Monoblepharis macrandra
Monoblepharis polymorpha
Hyaloraphidium curvatum
Monoblepharidomycetes
Neocallimastix sp.
Neocallimastix frontalis
Neocallimastigomycota
Entomophthora muscae
"Zygomycota, unresolved"
Rozella allomycis
Rozella
Drosophila melanogaster
Homo sapiens
Caenorhabditis elegans
Animalia
Dictyostelium discoideum
Populus trichocarpa
Phytophthora sojae
Toxoplasma gondii
Cryptosporidium parvum
Cyanidioschyzon merolae
Other eukaryotes (Outgroup)

Figure S1b

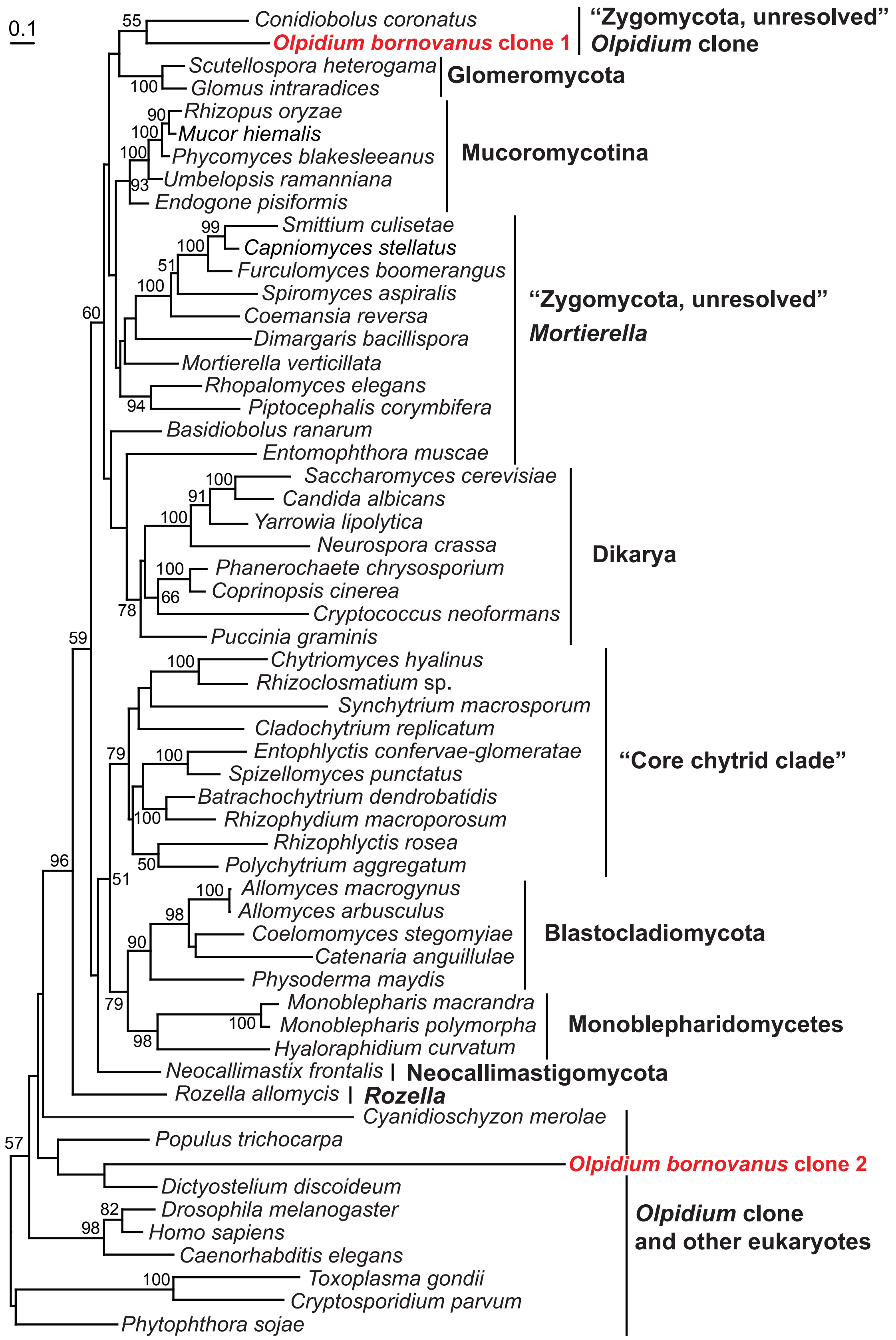

0.1
Conidiobolus coronatus
Olpidium bornovanus clone 1
"Zygomycota, unresolved"
Olpidium clone
Scutellospora heterogama
Glomus intraradices
Glomeromycota
Rhizopus oryzae
Mucor hiemalis
Phycomyces blakesleeanus
Umbelopsis ramanniana
Endogone pisiformis
Mucoromycotina
Smittium culisetae
Capniomyces stellatus
Furculomyces boomerangus
Spiromyces aspiralis
Coemansia reversa
Dimargaris bacillispora
Mortierella verticillata
Rhopalomyces elegans
Piptocephalis corymbifera
Basidiobolus ranarum
Entomophthora muscae
"Zygomycota, unresolved"
Mortierella
Saccharomyces cerevisiae
Candida albicans
Yarrowia lipolytica
Neurospora crassa
Phanerochaete chrysosporium
Coprinopsis cinerea
Cryptococcus neoformans
Puccinia graminis
Dikarya
Chytriomyces hyalinus
Rhizoclosmatium sp.
Synchytrium macrosporum
Cladochytrium replicatum
Entophlyctis confervae-glomeratae
Spizellomyces punctatus
Batrachochytrium dendrobatidis
Rhizophydium macroporosum
Rhizophlyctis rosea
Polychytrium aggregatum
"Core chytrid clade"
Allomyces macrogynus
Allomyces arbusculus
Coelomomyces stegomyiae
Catenaria anguillulae
Physoderma maydis
Blastocladiomycota
Monoblepharis macrandra
Monoblepharis polymorpha
Hyaloraphidium curvatum
Monoblepharidomycetes
Neocallimastix frontalis
Neocallimastigomycota
Rozella allomycis
Rozella
Cyanidioschyzon merolae
Populus trichocarpa
Olpidium bornovanus clone 2
Dictyostelium discoideum
Drosophila melanogaster
Homo sapiens
Caenorhabditis elegans
Toxoplasma gondii
Cryptosporidium parvum
Phytophthora sojae
Olpidium clone
and other eukaryotes
55
100
90
100
100
93
99
100
51
100
94
60
100
91
100
100
66
78
59
100
79
100
100
50
51
100
98
90
79
100
98
96
57
82
98
100

Figure S1c

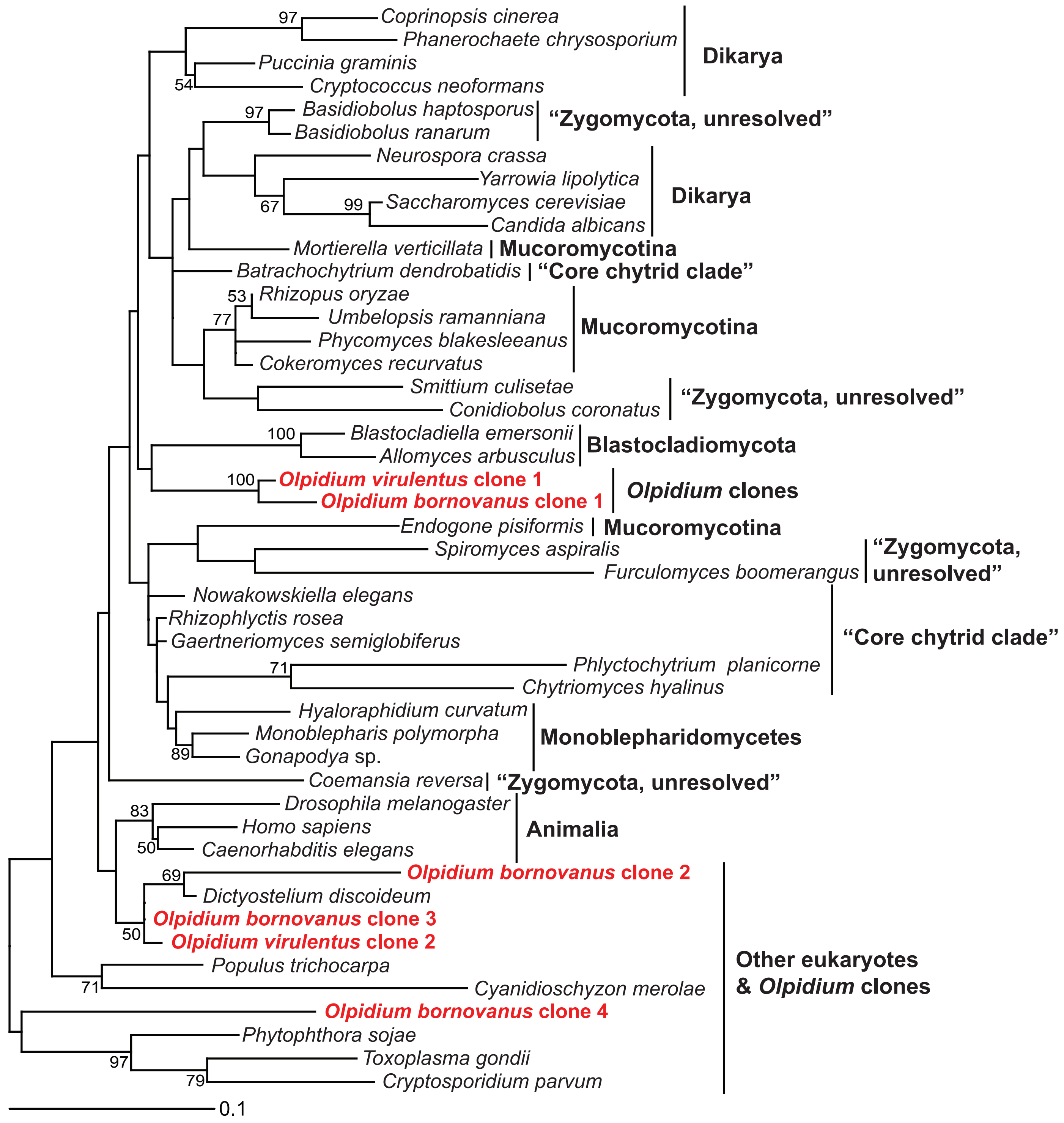
97
Coprinopsis cinerea
Phanerochaete chrysosporium
Dikarya
Puccinia graminis
54
Cryptococcus neoformans
97
Basidiobolus haptosporus
Basidiobolus ranarum
"Zygomycota, unresolved"
Neurospora crassa
Yarrowia lipolytica
Dikarya
99
Saccharomyces cerevisiae
67
Candida albicans
Mortierella verticillata | Mucoromycotina
Batrachochytrium dendrobatidis | "Core chytrid clade"
53
Rhizopus oryzae
77
Umbelopsis ramanniana
Mucoromycotina
Phycomyces blakesleeanus
Cokeromyces recurvatus
Smittium culisetae
"Zygomycota, unresolved"
Conidiobolus coronatus
100
Blastocladiella emersonii
Blastocladiomycota
Allomyces arbusculus
100
Olpidium virulentus clone 1
Olpidium clones
Olpidium bornovanus clone 1
Endogone pisiformis | Mucoromycotina
Spiromyces aspiralis
"Zygomycota, unresolved"
Furculomyces boomerangus
Nowakowskiella elegans
Rhizophlyctis rosea
"Core chytrid clade"
Gaertneriomyces semiglobiferus
71
Phlyctochytrium planicorne
Chytriomyces hyalinus
Hyaloraphidium curvatum
Monoblepharis polymorpha
Monoblepharidomycetes
89
Gonapodya sp.
Coemansia reversa | "Zygomycota, unresolved"
83
Drosophila melanogaster
Homo sapiens
Animalia
50
Caenorhabditis elegans
69
Olpidium bornovanus clone 2
Dictyostelium discoideum
Olpidium bornovanus clone 3
50
Olpidium virulentus clone 2
Other eukaryotes & Olpidium clones
Populus trichocarpa
71
Cyanidioschyzon merolae
Olpidium bornovanus clone 4
Phytophthora sojae
Toxoplasma gondii
97
79
Cryptosporidium parvum
0.1

Figure S1d
