# Supplementary material for: A multigene phylogeny of Olpidium and its implications for early fungal evolution
Source: BMC Evol Biol. 2011 Nov 15;11:331. doi: 10.1186/1471-2148-11-331 (PMC3247622; doi:10.1186/1471-2148-11-331)
Supplement: Additional file 2 — Figure S2 and Table S1. Figure S2. The phylogeny of the kingdom Fungi, including Rozella allomycis, based on likelihood analysis of amino acid sequences of four concatenated protein-encoding genes. Table S1. Tree topology tests showing that most of the alternative phylogenetic positions of Rozella could not be rejected. [file 1471-2148-11-331-S2.PDF]

Sekimoto *et al.* A multigene phylogeny of *Olpidium* and its implications for early fungal evolution.

Additional file 2

Figure S2. The phylogeny of the kingdom Fungi based on a four-protein concatenated dataset, including *Rozella* (63 OTUs, 2973 sites).

Maximum likelihood tree from thorough likelihood searches ("-f a" option) using the model that fitted best, LG+G+F. Numbers at the branches represent support from 1000 rapid bootstrap replicates. Searches were conducted on CIPRES Science Gateway Web server (with RAxML-HPC2 on Abe 7.2.7; Stamatakis et al. [49]). Two *Olpidium* species are indicated in *bold red*. The tree is similar to Figure 2 in the main article, but it includes *RPB1* and *RPB2* sequences of a unicellular endoparasitic chytrid, *Rozella allomycis*, which is located at the most basal position in the kingdom Fungi with bootstrap support of 67%. In the tree, four clades of chytrids (Blastocladiomycota + Neocallimastigomycota + Monoblepharidomycetes + "core chytrid clade") together formed a monophyletic group. This contrasts with Figure 2 where Blastocladiomycota and Monoblepharidomycetes were paraphyletic to the clade containing Neocallimastigomycota and the "core chytrid clade".

Table S1. Comparison of alternative possible phylogenetic positions of the chytrid fungus, *Rozella allomycis* using the Approximately Unbiased (AU) test and weighted Shimodaira-Hasegawa (wSH) test .

We compared alternative positions for *Rozella allomycis* using the AU test and the wSH test [52, 53], both implemented by CONSEL v0.20 [54], with site-wise likelihood values estimated in PAUP v.4.0b10 [55]. Each constrained tree was based on an initial guide tree with a single internal branch, generated in MacClade version 4.08 [56]. The most likely tree, given the constraint, was found using 100 search replicates in RAxML version 7.2.8 with the LG+G+F model, as conducted on the CIPRES Science Gateway Web server (on RAxML-HPC2 on TeraGrid) [49]. The AU test rejected *Rozella* forming a clade with Monoblepharidomycetes and with Mucoromycotina, but the more conservative wSH test did not reject any alternative topologies.

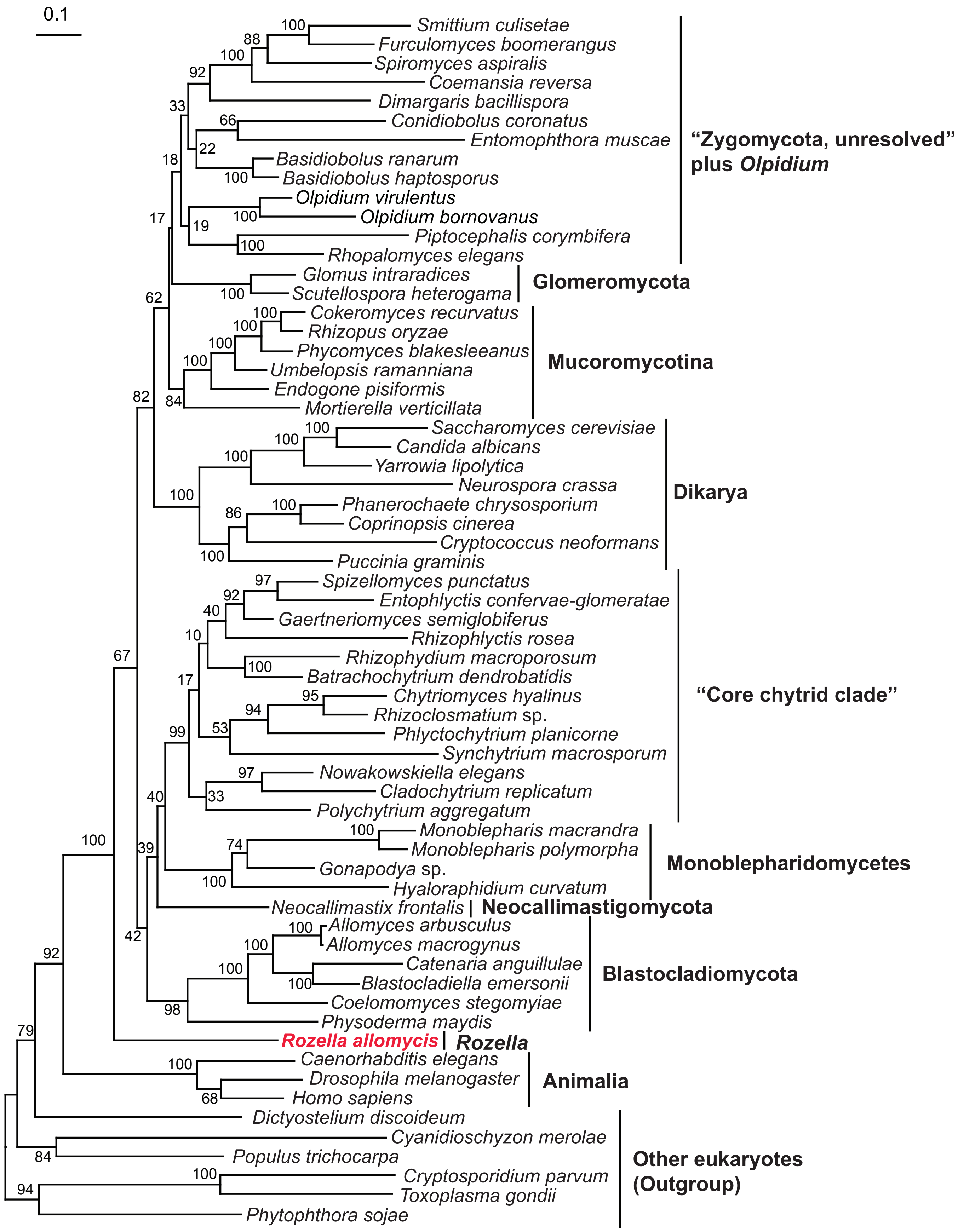
0.1
Smittium culisetae
Furculomyces boomerangus
Spiromyces aspiralis
Coemansia reversa
Dimargaris bacillispora
Conidiobolus coronatus
Entomophthora muscae
Basidiobolus ranarum
Basidiobolus haptosporus
Olpidium virulentus
Olpidium bornovanus
Piptocephalis corymbifera
Rhopalomyces elegans
"Zygomycota, unresolved" plus Olpidium
Glomus intraradices
Scutellospora heterogama
Glomeromycota
Cokeromyces recurvatus
Rhizopus oryzae
Phycomyces blakesleeanus
Umbelopsis ramanniana
Endogone pisiformis
Mortierella verticillata
Mucoromycotina
Saccharomyces cerevisiae
Candida albicans
Yarrowia lipolytica
Neurospora crassa
Phanerochaete chrysosporium
Coprinopsis cinerea
Cryptococcus neoformans
Puccinia graminis
Dikarya
Spizellomyces punctatus
Entophlyctis confervae-glomeratae
Gaertneriomyces semiglobiferus
Rhizophlyctis rosea
Rhizophydium macroporosum
Batrachochytrium dendrobatidis
Chytriomyces hyalinus
Rhizoclosmatium sp.
Phlyctochytrium planicorne
Synchytrium macrosporum
Nowakowskiella elegans
Cladochytrium replicatum
Polychytrium aggregatum
"Core chytrid clade"
Monoblepharis macrandra
Monoblepharis polymorpha
Gonapodya sp.
Hyaloraphidium curvatum
Monoblepharidomycetes
Neocallimastix frontalis
Neocallimastigomycota
Allomyces arbusculus
Allomyces macrogynus
Catenaria anguillulae
Blastocladiella emersonii
Coelomomyces stegomyiae
Physoderma maydis
Blastocladiomycota
Rozella allomycis
Rozella
Caenorhabditis elegans
Drosophila melanogaster
Homo sapiens
Animalia
Dictyostelium discoideum
Cyanidioschyzon merolae
Populus trichocarpa
Cryptosporidium parvum
Toxoplasma gondii
Phytophthora sojae
Other eukaryotes (Outgroup)

Figure S2

Table S1

Tree	Constraint	ΔlnL	AU[1]	wSH[1]
1	*Rozella* sister to all other fungi (Best tree, Figure S2)	0.0	0.773	0.936
2	*Rozella* united with Neocallimastigomycota	26.3	0.448	0.730
3	*Rozella* united with Blastocladiomycota	26.5	0.357	0.655
4	*Rozella* sister to all terrestrial fungi	29.3	0.383	0.716
5	*Rozella* united with "Core chytrid clade"	38.8	0.208	0.643
6	*Rozella* united with Dikarya	47.8	0.135	0.382
7	*Rozella* united with Monoblepharidomycetes	58.0	0.038*	0.281
8	*Rozella* united with Glomeromycota, in Zygomycota	60.2	0.072	0.251
9	*Rozella* united with "Zygomycota, unresolved"	71.8	0.054	0.187
10	*Rozella* united with Mucoromycotina, in Zygomycota	94.8	0.005**	0.050

[1]AU, Approximately Unbiased; wSH, weighted Shimodaira-Hasegawa test. The constrained tree was significantly worse than the best tree (Figure S2) at $P < 0.05$* or $P < 0.01$**.
