# Supplementary material for: A multigene phylogeny of Olpidium and its implications for early fungal evolution
Source: BMC Evol Biol. 2011 Nov 15;11:331. doi: 10.1186/1471-2148-11-331 (PMC3247622; doi:10.1186/1471-2148-11-331)
Supplement: Additional file 3 — Table S2. Species sequenced in this study, including voucher numbers and primer sets used. [file 1471-2148-11-331-S3.PDF]

Sekimoto *et al*. A multigene phylogeny of *Olpidium* and its implications for early fungal evolution.

Additional file 3

Table S2. The list of species sequenced in this study, including voucher numbers and primer set used. Classification in the table is based on Hibbett et al. [4]. The internal transcribed spacer (ITS) regions of *Olpidium virulentus* UBC F19784 (not on the list below) were amplified and sequenced with the primer set “SR11MF-LR3R”.

PHYLUM / Subphylum or PHYLUM/ Class	Classification labelled in Figures and Tables	Species name	Voucher #	*eukaryotic translation elongation factor 2*	*RNA polymerase II largest subunit*	*RNA polymerase II second largest subunit*	*actin*
SUBPHYLA INCERTAE SEDIS / Mucoromycotina	Mucoromycotina	*Mortierella verticillata*	NRRL 6337	12zext-Eu1453Ra, Rhiz1650-620zint	-	-	-
SUBPHYLA INCERTAE SEDIS / Mucoromycotina	Mucoromycotina	*Endogone pisiformis*	BL045	-	-	-	Act1G-Actjadr
SUBPHYLA INCERTAE SEDIS / Mucoromycotina	Mucoromycotina	*Umbelopsis ramanniana*	NRRL 5844	12zext-Eu1453Rb, Eu453-620zint	-	-	-
SUBPHYLA INCERTAE SEDIS / Mucoromycotina	Mucoromycotina	*Cokeromyces recurvatus*	BL061	24bext-620bint	-	-	-
SUBPHYLA INCERTAE	"Zygomycota,	*Coemansia*	NRRL1564	12zext-Eu1453Rb,	-	-	Act1G-ActjR

SEDIS / Kickxellomycotina	unresolved"	*reversa*		Spiz453-620zint			
SUBPHYLA INCERTAE SEDIS / Kickxellomycotina	"Zygomycota, unresolved"	*Spiromyces aspiralis*	NRRL22631	12zext-Eu1648Rb, Eu453-620zint	-	-	Act1G-ActjR
SUBPHYLA INCERTAE SEDIS / Kickxellomycotina	"Zygomycota, unresolved"	*Furculomyces boomerangus*	BL059	12zext-620zint	-	-	ActjFqk-Actjtfq
SUBPHYLA INCERTAE SEDIS / Kickxellomycotina	"Zygomycota, unresolved"	*Smittium culisetae*	BL023	12zext-620zint	-	-	Act1G-ActjR
SUBPHYLA INCERTAE SEDIS / Entomophthoromycotina	"Zygomycota, unresolved"	*Conidiobolus coronatus*	NRRL28638	12zext-620zint, Eu265F-Eu2255R	-	-	Act1G-ActjR
BLASTOCLADIOMYCOTA / Blastocladiomycetes	Blastocladiomycota	*Blastocladiella emersonii*	BL058	12zext-Eu1453Ra, Spiz453-620zint	-	-	-
BLASTOCLADIOMYCOTA / Blastocladiomycetes	Blastocladiomycota	*Catenaria anguillulae*	PL 171	12zext-620bint, Eu554F-Eu1531R	-	-	-
BLASTOCLADIOMYCOTA / Blastocladiomycetes	Blastocladiomycota	*Allomyces arbusculus*	BL002	12zext-Eu1453Rb, Eu453-620zint	-	-	Act1G-ActjR
CHYTRIDIOMYCOTA / Chytridiomycetes	"Core chytrid clade"	*Phlyctochytrium planicorne*	JEL47, BL062	24bext-Eu1453Ra, Spiz453-620zint	-	-	Act1G-ActjR
CHYTRIDIOMYCOTA / Chytridiomycetes	"Core chytrid clade"	*Chytriomyces hyalinus*	BR 097	12zext-620zint	-	-	ActjFqk-ActjR
CHYTRIDIOMYCOTA / Chytridiomycetes	"Core chytrid clade"	*Gaertneriomyces semiglobiferus*	BL035	64fin2F-Eu1453Ra, Spiz453-620zint	-	-	Act1G-ActjR

CHYTRIDIOMYCOTA / Chytridiomycetes	"Core chytrid clade"	*Spizellomyces punctatus*	BR117A	12zext-Eu1039R, Eu453-620zint	-	-	-
CHYTRIDIOMYCOTA / Chytridiomycetes	"Core chytrid clade"	*Cladochytrium replicatum*	JEL180	12zext-620zint, 12zext-Eu535R	-	-	-
CHYTRIDIOMYCOTA / Chytridiomycetes	"Core chytrid clade"	*Nowakowskiella elegans*	BR 416	12zext-Eu1504R, Eu933F-620zint	-	-	Act1G-ActjR
CHYTRIDIOMYCOTA / Monoblepharidomycetes	Monoblepharidomycetes	*Hyaloraphidium curvatum*	SAG 235-1,BL011	-	-	-	ActjFqk-ActjR
CHYTRIDIOMYCOTA / Monoblepharidomycetes	Monoblepharidomycetes	*Monoblepharis polymorpha*	JEL 486	12zext-Eu1453Rb, Eu227F-620bint	-	-	Act1-ActjR
CHYTRIDIOMYCOTA / Monoblepharidomycetes	Monoblepharidomycetes	*Gonapodya* sp.	JEL183	12zext-620bint, Eu227F-620bint	-	-	Act1G-ActjR
GENUS INCERTAE SEDIS	"Zygomycota, unresolved"	*Basidiobolus ranarum*	NRRL34594, BL017	12zext-620zint	-	-	ActjFqk-ActjR
GENUS INCERTAE SEDIS	"Zygomycota, unresolved"	*Basidiobolus haptosporus*	NRRL 28635	12zext-Eu1453Rb, Eu453-620zint	RPB1Ac-RPB1Fr, RPB1831F-RPB11154R, RPB1823F-RPB11154R	-	ActjFqk-ActjR
GENUS INCERTAE SEDIS	"Zygomycota, unresolved"	*Olpidium bornovanus*	UBC F19785	Spiz453-620zint	RPB1Df-RPB1Fr	RPB2-214F - RPB2-581R, RPB2-716F - RPB2-1019R	Actjkiw-Actjkys
GENUS INCERTAE SEDIS	"Zygomycota,	*Olpidium*	UBC F19784	12zext-620zint	RPB1Df-RPB1Fr	-	Actjkiw-Actjkys

	unresolved"	*virulentus*					
GENUS INCERTAE SEDIS	"Core chytrid clade"	*Rhizophlyctis rosea*	BR186	12zext-620zint	-	-	Act1G-ActjR
CHYTRIDIOMYCOTA / Chytridiomycetes	"Core chytrid clade"	*Entophlyctis helioformis*	JEL326, BL041	125bint-Eu1453Ra, Eu554F-620zint	-	-	-
CHYTRIDIOMYCOTA / Chytridiomycetes	"Core chytrid clade"	*Rhizophydium globosum*	JEL 222	12zext-620zint, 125bint-Eu1066R	-	-	-
GENUS INCERTAE SEDIS	*Olpidium* clone(s)	*Olpidium virulentus* clone 2 (Ef-2 paralog?)	UBC F19784	12zext-620zint	-	-	-
GENUS INCERTAE SEDIS	*Olpidium* clone(s)	*Olpidium bornovanus* clone 2 (Ef-2 paralog?)	UBC F19785	12zext-620zint	-	-	-
GENUS INCERTAE SEDIS	*Olpidium* clone(s)	*Olpidium bornovanus* clone 2 (RPB2 paralog?)	UBC F19785	-	-	RPB2-716F - RPB2-1019R	-
GENUS INCERTAE SEDIS	*Olpidium* clone(s)	*Olpidium bornovanus* clone 2 (actin paralog?)	UBC F19785	-	-	-	Actjkiw-Actjkys

GENUS INCERTAE SEDIS	*Olpidium* clone(s)	*Olpidium bornovanus* clone 3 (actin paralog?)	UBC F19785	-	-	-	Actjfqk-Actjtfq
GENUS INCERTAE SEDIS	*Olpidium* clone(s)	*Olpidium bornovanus* clone 4 (actin paralog?)	UBC F19785	-	-	-	Act1G-ActjR
GENUS INCERTAE SEDIS	*Olpidium* clone(s)	*Olpidium virulentus* clone 2 (actin paralog?)	UBC F19784	-	-	-	Act1G-ActjR

Voucher numbers.

NRRL: ARS Culture Collection (http://nrrl.ncaur.usda.gov/)

BL: UC Berkeley Microgarden

PL: A collection from Peter M. Letcher (University of Alabama)

BR: A collection from Donald J. S. Barr, available from Canadian Collection of Fungal Cultures (CCFC) (http://wdcm.nig.ac.jp/CCINFO/CCINFO.xml?150)

JEL: A collection from Joyce E. Longcore (University of Maine)

SAG: Sammulung von Algenkulturen Göttingen, University of Göttingen (http://epsag.uni-goettingen.de)

UBC: UBC Herbarium, University of British Columbia (http://www.beatymuseum.ubc.ca/herbarium/index.html)
