# Supplementary material for: A multigene phylogeny of Olpidium and its implications for early fungal evolution
Source: BMC Evol Biol. 2011 Nov 15;11:331. doi: 10.1186/1471-2148-11-331 (PMC3247622; doi:10.1186/1471-2148-11-331)
Supplement: Additional file 4 — Table S3. Primers used in this study. [file 1471-2148-11-331-S4.PDF]

Sekimoto *et al*. A multigene phylogeny of *Olpidium* and its implications for early fungal evolution.

Additional file 4

Table S3. The list of primers used in this study. James et al. [5] and Hoffmann et al. [45] can be found at refernce list in the main article. An unpublished primer “EFT2_2F” [0] was kindly provided by Bernard Ball (Lutzoni Lab, Duke University, USA).

Primer name	Gene	Sequence (5' to 3')	Reference
12zext	*Ef-2*	GTCATTGCTCACGTCGACCACGG	This study
125bint	*Ef-2*	TGTGTCCAAACCGAGACTGT	This study
620zint	*Ef-2*	TCGGGACCGAAACACCAGATCTT	This study
24bext	*Ef-2*	GTCATTGCTCACGTCGATCATGG	This study
620bint	*Ef-2*	TCGGGACCGAAACACCAAATCTT	This study
24bext	*Ef-2*	GTCATTGCTCACGTCGATCATGG	This study
EFT2_2F	*Ef-2*	GCBCAYGTYGAYCAYGG	This study [0]
Eu227F	*Ef-2*	ATCAAGTCCACGGCCATTTC	This study
Eu265F	*Ef-2*	TTACCTGAAGAAGCTATGGTTG	This study
Eu453	*Ef-2*	CAAGGTTGATCGTGCTCTTCTCG	This study
Eu535R	*Ef-2*	GTTTCGGTCTGCACACACAC	This study
Eu554F	*Ef-2*	GTGTGTGTGCAGACCGAGAC	This study
Eu933F	*Ef-2*	CTTTCGGTTCTGGTCTGCAC	This study
Eu1039R	*Ef-2*	CTTGGTCTTGGGGTTGAAGTAGTT	This study
Eu1066R	*Ef-2*	CTTGGTCTTAGGGTTGAAGTAGTT	This study
Eu1453Ra	*Ef-2*	TCGGAGGTGGGAACCATCTT	This study
Eu1453Rb	*Ef-2*	TCGGAGGTTGGCACCATCTT	This study
Eu1504R	*Ef-2*	AATCGACCCTTGTCCGAAGT	This study
Eu1531R	*Ef-2*	CCAGAGAAGACACGACCGAA	This study
Eu2255R	*Ef-2*	GACTAAATGACGACCACGAG	This study
Rhiz1650	*Ef-2*	ACAAGGTTCTCGGTGACATCATG	This study

Spiz453	*Ef-2*	CAAGGTTGACCGTGCCCTGCTCG	This study
RPB1823F	*RPB1*	ATGTCYGCTTGTGTYGGTCAACA	This study
RPB1831F	*RPB1*	YGTCGARGGAAAGCGDATYCC	This study
RPB11154R	*RPB1*	GGAAHTCYTCMADVACVCGCTT	This study
RPB1Ac	*RPB1*	GARTGYCCDGGDCAYTTYGG	James et al. [5]
RPB1Df	*RPB1*	TACAATGCYGAYTTYGAYGG	James et al. [5]
RPB1Fr	*RPB1*	CCYTCNCKWCCWCCCATDGCRTG	James et al. [5]
RPB2-214F	*RPB2*	GAYGAYMGNGAYCAYWWYGG	This study
RPB2-581R	*RPB2*	CGAGTCTGCCAGGTCTTCCG	This study
RPB2-716F	*RPB2*	GGNTAYAAYCARGARGA	This study
RPB2-1019R	*RPB2*	ATYTTRTCRTCNACCATRTG	This study
Act1	*actin*	TGGGACGATATGGAIAAIATCTGGCA	Hoffmann et al. [45]
Act1G	*actin*	TGGGACGATATGGAGAAGATCTGGCA	This study
Actjadr	*actin*	TCCTTTTGCATACGGTCRGC	This study
ActjR	*actin*	TCGTATTCCTGCTTTGAGATCCA	This study
Actjtfq	*actin*	CCACATTTGTTGGAARGTAG	This study
ActjFqk	*actin*	ATGGGTCAAAAGGACTC	This study
Actjkiw	*actin*	AGAAGATCTGGCACCAC	This study
Actjkys	*actin*	GATCCAGACAGAGTACTTTC	This study
SR11MF	*SSU rRNA*	CCGTCGCTACTACCGATTGAATG	This study
LR3R	*LSU rRNA*	TCACGGTACTTGTTYGCTATCGG	This study
