# Supplementary material for: A multigene phylogeny of Olpidium and its implications for early fungal evolution
Source: BMC Evol Biol. 2011 Nov 15;11:331. doi: 10.1186/1471-2148-11-331 (PMC3247622; doi:10.1186/1471-2148-11-331)
Supplement: Additional file 5 — Table S4. GenBank accession numbers of sequences used in this study. [file 1471-2148-11-331-S5.PDF]

Sekimoto *et al.* A multigene phylogeny of *Olpidium* and its implications for early fungal evolution.

Additional file 5

Table S4. GenBank accession numbers of gene sequences used in this study. Classification is based on Hibbett et al. [4]. The accession number of the internal transcribed spacer regions of *Olpidium virulentus* UBC F19784, sequenced to verify identity, is AB625456.

Sources of sequences: *Grey cells*, sequences determined in this study. [A] Whole genome data or EST libraries, the Broad Institute (http://www.broadinstitute.org/). [B] DOE Joint Genome Institute (http://www.jgi.doe.gov/). [C] Alignments from James et al. [5], from Assembling the Fungal Tree of Life (http://aftol.org/). [D] Alignment from Voigt & Wöstemeyer [33]. References are listed in full in the main article.

Fungal taxa

PHYLUM / Subphylum or PHYLUM/ Class	Classification labelled in Figures and Tables	Species name	*eukaryotic translation elongation factor 2*	*RNA polymerase II largest subunit*	*RNA polymerase II second largest subunit*	*actin*
BASIDIOMYCOTA / Pucciniomycotina	Dikarya	*Puccinia graminis*	[A] PGTG_07969.2	-	[A] PTTG_03658.1	EZ116217
BASIDIOMYCOTA / Agaricomycotina	Dikarya	*Coprinopsis cinerea*	XM_001829285	[C]	[C]	XM_001839313
BASIDIOMYCOTA / Agaricomycotina	Dikarya	*Phanerochaete chrysosporium*	[B] scaffold_13 (972239-975048)	[C]	[C]	AB115328
BASIDIOMYCOTA / Agaricomycotina	Dikarya	*Cryptococcus neoformans*	XM_570549	[C]	[C]	[A] *C. neoformans* Serotype B (CNBG_1429)
ASCOMYCOTA / Pezizomycotina	Dikarya	*Neurospora crassa*	XM_957193	[C]	[C]	U78026
ASCOMYCOTA / Saccharomycotina	Dikarya	*Yarrowia lipolytica*	XM_499612	[C]	[C]	AJ250347
ASCOMYCOTA / Saccharomycotina	Dikarya	*Saccharomyces cerevisiae*	M59370	[C]	[C]	V01288
ASCOMYCOTA / Saccharomycotina	Dikarya	*Candida albicans*	AF107286	[C]	[C]	X16377.1
GLOMEROMYCOTA / Glomeromycetes	Glomeromycota	*Scutellospora heterogama*	-	[C]	[C]	-
GLOMEROMYCOTA / Glomeromycetes	Glomeromycota	*Glomus intraradices*	-	[C]	[C]	-

SUBPHYLA INCERTAE SEDIS / Mucoromycotina	Mucoromycotina	*Mortierella verticillata*	AB609150	[C]	[C]	[D]
SUBPHYLA INCERTAE SEDIS / Mucoromycotina	Mucoromycotina	*Endogone pisiformis*	-	[C]	[C]	AB609182
SUBPHYLA INCERTAE SEDIS / Mucoromycotina	Mucoromycotina	*Umbelopsis ramanniana*	AB609151	[C]	[C]	[D]
SUBPHYLA INCERTAE SEDIS / Mucoromycotina	Mucoromycotina	*Phycomyces blakesleeanus*	[B] scaffold_23 (574778-577453)	[C]	[C]	[D]
SUBPHYLA INCERTAE SEDIS / Mucoromycotina	Mucoromycotina	*Cokeromyces recurvatus*	AB609154	-	-	[D]
SUBPHYLA INCERTAE SEDIS / Mucoromycotina	Mucoromycotina	*Rhizopus oryzae*	[A] RO3G_12127	[C]	[C]	[A] RO3G_14002.3
SUBPHYLA INCERTAE SEDIS / Kickxellomycotina	"Zygomycota, unresolved"	*Dimargaris bacillispora*	-	[C]	[C]	-
SUBPHYLA INCERTAE SEDIS / Kickxellomycotina	"Zygomycota, unresolved"	*Coemansia reversa*	AB609161	[C]	[C]	AB609183
SUBPHYLA INCERTAE SEDIS / Kickxellomycotina	"Zygomycota, unresolved"	*Spiromyces aspiralis*	AB609155	[C]	[C]	HM117710
SUBPHYLA INCERTAE SEDIS / Kickxellomycotina	"Zygomycota, unresolved"	*Furculomyces boomerangus*	AB609157	EF014380	EF014397	HM117714
SUBPHYLA INCERTAE SEDIS / Kickxellomycotina	"Zygomycota, unresolved"	*Smittium culisetae*	AB609159	EF014378	EF014395	HM117719

SUBPHYLA INCERTAE SEDIS / Entomophthoromycotina	"Zygomycota, unresolved"	*Conidiobolus coronatus*	AB609152	[C]	[C]	HM117709
SUBPHYLA INCERTAE SEDIS / Entomophthoromycotina	"Zygomycota, unresolved"	*Entomophthora muscae*	-	[C]	[C]	-
SUBPHYLA INCERTAE SEDIS / Zoopagomycotina	"Zygomycota, unresolved"	*Piptocephalis corymbifera*	-	[C]	[C]	-
SUBPHYLA INCERTAE SEDIS / Zoopagomycotina	"Zygomycota, unresolved"	*Rhopalomyces elegans*	-	[C]	[C]	-
BLASTOCLADIOMYCOTA / Blastocladiomycetes	Blastocladiomycota	*Physoderma maydis*	-	[C]	[C]	-
BLASTOCLADIOMYCOTA / Blastocladiomycetes	Blastocladiomycota	*Coelomomyces stegomyiae*	-	[C]	[C]	-
BLASTOCLADIOMYCOTA / Blastocladiomycetes	Blastocladiomycota	*Blastocladiella emersonii*	AB609153	EF014387	-	AY582842
BLASTOCLADIOMYCOTA / Blastocladiomycetes	Blastocladiomycota	*Catenaria anguillulae*	AB609173	EF014386	EF014405	-
BLASTOCLADIOMYCOTA / Blastocladiomycetes	Blastocladiomycota	*Allomyces arbusculus*	AB609160	[C]	[C]	HM117708
BLASTOCLADIOMYCOTA / Blastocladiomycetes	Blastocladiomycota	*Allomyces macrogynus*	-	EF014385	EF014404	-
CHYTRIDIOMYCOTA / Chytridiomycetes	"Core chytrid clade"	*Phlyctochytrium planicorne*	AB609162	-	-	HM117703

CHYTRIDIOMYCOTA / Chytridiomycetes	"Core chytrid clade"	*Chytriomyces hyalinus*	AB609172	EF014393	EF014409	HM117702
CHYTRIDIOMYCOTA / Chytridiomycetes	"Core chytrid clade"	*Rhizoclosmatium sp.*	-	[C]	[C]	-
CHYTRIDIOMYCOTA / Chytridiomycetes	"Core chytrid clade"	*Polychytrium aggregatum*	-	[C]	[C]	-
CHYTRIDIOMYCOTA / Chytridiomycetes	"Core chytrid clade"	*Entophlyctis confervae-glomeratae*	-	EF014389	EF014407	-
CHYTRIDIOMYCOTA / Chytridiomycetes	"Core chytrid clade"	*Gaertneriomyces semiglobiferus*	AB609167	-	-	HM117705
CHYTRIDIOMYCOTA / Chytridiomycetes	"Core chytrid clade"	*Spizellomyces punctatus*	AB609171	[C]	[C]	-
CHYTRIDIOMYCOTA / Chytridiomycetes	"Core chytrid clade"	*Rhizophydium macroporosum*	-	[C]	[C]	-
CHYTRIDIOMYCOTA / Chytridiomycetes	"Core chytrid clade"	*Batrachochytrium dendrobatidis*	[B] scaffold_1 (590109-593319)	[C]	[C]	[A] BDEG_03011
CHYTRIDIOMYCOTA / Chytridiomycetes	"Core chytrid clade"	*Synchytrium macrosporum*	-	[C]	[C]	-
CHYTRIDIOMYCOTA / Chytridiomycetes	"Core chytrid clade"	*Cladochytrium replicatum*	AB609168	[C]	[C]	-
CHYTRIDIOMYCOTA / Chytridiomycetes	"Core chytrid clade"	*Nowakowskiella elegans*	AB609166	-	-	HM117701
NEOCALLIMASTIGOMYCOTA / Neocallimastigomycetes	Neocallimastigomycota	*Neocallimastix frontalis*	-	EF014394	EF014412	-
CHYTRIDIOMYCOTA / Monoblepharidomycetes	Monoblepharidomycetes	*Hyaloraphidium curvatum*	-	[C]	[C]	HM117715

CHYTRIDIOMYCOTA / Monoblepharidomycetes	Monoblepharidomycetes	*Monoblepharis macrandra*	-	AF315822	EF014410	-
CHYTRIDIOMYCOTA / Monoblepharidomycetes	Monoblepharidomycetes	*Monoblepharis polymorpha*	AB609165	[C]	[C]	HM117707
CHYTRIDIOMYCOTA / Monoblepharidomycetes	Monoblepharidomycetes	*Gonapodya* sp.	AB609164	-	-	HM117706
GENUS INCERTAE SEDIS	"Zygomycota, unresolved"	*Basidiobolus ranarum*	AB609158	[C]	[C]	HM117716
GENUS INCERTAE SEDIS	"Zygomycota, unresolved"	*Basidiobolus haptosporus*	AB609156	AB609178		HM117717
GENUS INCERTAE SEDIS	"Zygomycota, unresolved"	*Olpidium bornovanus*	AB609174 (clone 1)	AB609179	AB609180 (clone 1)	AB609184 (clone 1)
GENUS INCERTAE SEDIS	"Zygomycota, unresolved"	*Olpidium virulentus*	AB609176 (clone 1)	[C]	-	HM117711 (clone 1)
GENUS INCERTAE SEDIS	*Rozella*	*Rozella allomycis*	-	[C]	[C]	-
GENUS INCERTAE SEDIS	"Core chytrid clade"	*Rhizophlyctis rosea*	AB609170	[C]	[C]	HM117704

Non-fungal taxa

KINGDOM	Classification labelled in Figures and Tables	Species name	*eukaryotic translation elongation factor 2*	*RNA polymerase II largest subunit*	*RNA polymerase II second largest subunit*	*actin*
ANIMALIA	Animalia, or Other eukaryotes	*Caenorhabditis elegans*	Z81068	[C]	[C]	X16796
ANIMALIA	Animalia, or Other eukaryotes	*Drosophila melanogaster*	NM_080366	[C]	[C]	[D]
ANIMALIA	Animalia, or Other eukaryotes	*Homo sapiens*	AY942181	[C]	[C]	[D]
MYCETOZOA	Other eukaryotes, or *Dictyostelium*	*Dictyostelium discoideum*	XM_631959	[C]	[C]	XM_630996
APICOMPLEXA	Other eukaryotes	*Toxoplasma gondii*	XM_002367737	[C]	[C]	XM_002369622
APICOMPLEXA	Other eukaryotes	*Cryptosporidium parvum*	XM_627193	[C]	[C]	M86241
CHROMISTA	Other eukaryotes	*Phytophthora sojae*	[B] scaffold_81 (112251-114827)	[C]	[C]	[B] scaffold_49 (280023-281156)
RHODOPHYTA	Other eukaryotes	*Cyanidioschyzon merolae*	AB095183	[C]	[C]	AB095179
PLANTAE	Other eukaryotes	*Populus trichocarpa*	XM_002310651	[C]	[C]	EF144294

The nucleotide sequences not included in the concatenated tree (Figures 2 and S2) but included in the trees of each individual gene data set (Additional file 4, Figure S1a-S1d)

PHYLUM / Subphylum or PHYLUM/ Class	Classification labelled in Figures and Tables	Species name	*eukaryotic translation elongation factor 2*	*RNA polymerase II largest subunit*	*RNA polymerase II second largest subunit*	*actin*
CHYTRIDIOMYCOTA / Chytridiomycetes	"Core chytrid clade"	*Entophlyctis helioformis*	AB609169	-	-	-
CHYTRIDIOMYCOTA / Chytridiomycetes	"Core chytrid clade"	*Rhizophydium globosum*	AB609163	-	-	HM117718 (we sequenced but not included in this study)
GENUS INCERTAE SEDIS	*Olpidium* clone(s)	*Olpidium virulentus* clone 2 (Ef-2 paralog?)	AB609177	-	-	-
GENUS INCERTAE SEDIS	*Olpidium* clone(s)	*Olpidium bornovanus* clone 2 (Ef-2 paralog?)	AB609175	-	-	-
GLOMEROMYCOTA / Glomeromycetes	Glomeromycota	*Glomus mosseae*	-	DQ294592	-	-
NEOCALLIMASTIGOMYCOTA / Neocallimastigomycetes	Neocallimastigomycota	*Neocallimastix* sp.	-	DQ294611	-	-
SUBPHYLA INCERTAE SEDIS / Mucoromycotina	Mucoromycotina	*Mucor hiemalis*	-	-	EF014398	-
SUBPHYLA INCERTAE SEDIS /	"Zygomycota,	*Capniomyces stellatus*	-	-	EF014396	-

Kickxellomycotina	unresolved"					
GENUS INCERTAE SEDIS	*Olpidium* clone(s)	*Olpidium bornovanus* clone 2 (RPB2 paralog?)	-	-	AB609181	-
GENUS INCERTAE SEDIS	*Olpidium* clone(s)	*Olpidium bornovanus* clone 2 (actin paralog?)	-	-	-	AB609185
GENUS INCERTAE SEDIS	*Olpidium* clone(s)	*Olpidium bornovanus* clone 3 (actin paralog?)	-	-	-	HM117712
GENUS INCERTAE SEDIS	*Olpidium* clone(s)	*Olpidium bornovanus* clone 4 (actin paralog?)	-	-	-	AB609186
GENUS INCERTAE SEDIS	*Olpidium* clone(s)	*Olpidium virulentus* clone 2 (actin paralog?)	-	-	-	HM117713
