# Supplementary material for: A multigene phylogeny of Olpidium and its implications for early fungal evolution
Source: BMC Evol Biol. 2011 Nov 15;11:331. doi: 10.1186/1471-2148-11-331 (PMC3247622; doi:10.1186/1471-2148-11-331)
Supplement: Additional file 6 — The initial guide trees with a single internal branch, and the most likely trees given the constraint, that we used for the tree topology tests in Table 1. All trees are in Newick format. [file 1471-2148-11-331-S6.DOC]

Sekimoto *et al*. A multigene phylogeny of *Olpidium* and its implications for early fungal evolution.

**Additional file 6**

The initial guide trees with a single internal branch and the most likely trees given as the constraint, that we used for tree topology test in Table 1. Trees are shown in Newick format. The numbering of the tree corresponds to the ones on Table 1.

**Tree labels**

AllomyRoze: ***Allomyces arbusculus***

Allommacr2: ***Allomyces macrogynus***

Bashaptosp: ***Basidiobolus haptosporus***

Basranarum: ***Basidiobolus ranarum***

Batrachoch: ***Batrachochytrium dendrobatidis***

Blastoclad: ***Blastocladiella emersonii***

Caeelegans: ***Caenorhabditis elegans***

Candidaalb: ***Candida albicans***

Catenaria1: ***Catenaria anguillulae***

Chytriohya: ***Chytriomyces hyalinus***

Cladochytr: ***Cladochytrium replicatum***

coelomomyc: ***Coelomomyces stegomyiae***

Coemansia2: ***Coemansia reversa***

Cokeromyce: ***Cokeromyces recurvatus***

Conidiobol: ***Conidiobolus coronatus***

Coprinopsi: ***Coprinopsis cinerea***

Cryptococu: ***Cryptococcus neoformans***

Cryptospor: ***Cryptosporidium parvum***

Cyanidiosi: ***Cyanidioschyzon merolae***

Dictyostel: ***Dictyostelium discoideum***

dimargaris: ***Dimargaris bacillispora***

Drosophila: ***Drosophila melanogaster***

endogonepi: ***Endogone pisiformis***

entomophth: ***Entomophthora muscae***

entophcon2: ***Entophlyctis confervae glomeratae***

Furculomyc: ***Furculomyces boomerangus***

Gaertnerio: ***Gaertneriomyces semiglobiferus***

glomusintr: ***Glomus intraradices***

Gonapodyas: ***Gonapodya* sp. JEL183**

Homosapien: ***Homo sapiens***

hyaloraphi: ***Hyaloraphidium curvatum***

Monoblema2: ***Monoblepharis macrandra***

Monoblepha: ***Monoblepharis polymorpha***

Mortierell: ***Mortierella verticillata***

neocallif2: ***Neocallimastix frontalis***

Neurospora: ***Neurospora crassa***

Nowakowski: ***Nowakowskiella elegans***

OlpbornoJD: ***Olpidium bornovanus***

Olpbrassi1: ***Olpidium virulentus***

Phanerocha: ***Phanerochaete chrysosporium***

Phlyctochy: ***Phlyctochytrium planicorne***

Phycomyces: ***Phycomyces blakesleeanus***

physoderma: ***Physoderma maydis***

Phytophsoj: ***Phytophthora sojae***

piptocepha: ***Piptocephalis corymbifera***

polychytri: ***Polychytrium aggregatum***

Populustri: ***Populus trichocarpa***

Pucciniagr: ***Puccinia graminis***

rhizoclosm: ***Rhizoclosmatium* sp. JEL347 h**

Rhizophlyc: ***Rhizophlyctis rosea***

rhizophmac: ***Rhizophydium macroporosum***

Rhizopusor: ***Rhizopus oryzae***

rhopalomyc: ***Rhopalomyces elegans***

Saccharomy: ***Saccharomyces cerevisiae***

scutellosp: ***Scutellospora heterogama***

Smittiumcu: ***Smittium culisetae***

Spiromyces: ***Spiromyces aspiralis***

Spizellomy: ***Spizellomyces punctatus***

synchytriu: ***Synchytrium macrosporum***

Toxoplasma: ***Toxoplasma gondii***

Umbelopsis: ***Umbelopsis ramanniana***

Yarrowiali: ***Yarrowia lipolytica***

**Tree 2 (*Olpidium* united with Glomeromycota in Zygomycota) Initial guide tree**

((OlpbornoJD,Olpbrassi1,scutellosp,glomusintr),(Pucciniagr,Coprinopsi,Phanerocha,Cryptococu,Neurospora,Yarrowiali,Saccharomy,Candidaalb,Mortierell,endogonepi,Umbelopsis,Phycomyces,Cokeromyce,Rhizopusor,dimargaris,Coemansia2,Spiromyces,Furculomyc,Smittiumcu,Conidiobol,entomophth,Basranarum,Bashaptosp,piptocepha,rhopalomyc,physoderma,coelomomyc,Blastoclad,Catenaria1,AllomyRoze,Allommacr2,neocallif2,Phlyctochy,Chytriohya,rhizoclosm,Rhizophlyc,polychytri,entophcon2,Gaertnerio,Spizellomy,rhizophmac,Batrachoch,synchytriu,Cladochytr,Nowakowski,hyaloraphi,Monoblema2,Monoblepha,Gonapodyas,Caeelegans,Drosophila,Homosapien,Dictyostel,Toxoplasma,Cryptospor,Phytophsoj,Cyanidiosi,Populustri));

**Tree 2 (*Olpidium* united with Glomeromycota in Zygomycota) Constraint**

(((((((((synchytriu:0.48064169452818739092,(Phlyctochy:0.26709310543147141148,(rhizoclosm:0.10154404251195760234,Chytriohya:0.14343209993888231257):0.13437409720054058204):0.08528682590191627444):0.07430939995777244289,((rhizophmac:0.21690514266822252809,Batrachoch:0.13358618526530166570):0.08492655007431178238,(Rhizophlyc:0.42208018526313584307,((entophcon2:0.22349702446302016634,Spizellomy:0.09109500222251191992):0.07857343571433852980,Gaertnerio:0.06687260088778561318):0.04121707953883586850):0.04412724132031863034):0.01861131786358100407):0.02337271706067341887,((Cladochytr:0.26308787150844298308,Nowakowski:0.12021118694603825927):0.12921649788201064468,polychytri:0.23757432809880701940):0.04221655102541393284):0.06685240798746253099,neocallif2:0.25625303062608428828):0.01411674274964827211,((hyaloraphi:0.36048249283123157127,(Gonapodyas:0.16563436439035866821,(Monoblema2:0.08941955617836050418,Monoblepha:0.05987285364473730886):0.30839749783564918140):0.03257343194161360644):0.13832051263515016126,((Caeelegans:0.22586796759491614695,(Drosophila:0.18673061917946176536,Homosapien:0.14601385313191053705):0.05662641931268820594):0.31150737660229010917,((((Cryptospor:0.40480258338732844070,Toxoplasma:0.39988629920778534510):0.42216595571554521582,Phytophsoj:0.47368180748809479708):0.10969108673389053876,(Cyanidiosi:0.76608717412466953434,Populustri:0.38514020697600370724):0.08757854128346141687):0.04006095353603175702,Dictyostel:0.48153798640510914186):0.06212733026308377149):0.15301215736142825818):0.02898769993501067871):0.02544283160366810070,((coelomomyc:0.18293707189014504766,((AllomyRoze:0.01188447023121346965,Allommacr2:0.00364017147070759507):0.11195142373764270516,(Catenaria1:0.20638780321833205544,Blastoclad:0.10643447651438585344):0.09272271983412180840):0.05747662705284382889):0.14247050150077866792,physoderma:0.30047037210603616542):0.09569410622651090825):0.04487266742512432266,((((dimargaris:0.37227181421526145400,(Coemansia2:0.40309548458474814270,((Furculomyc:0.14953238717979860928,Smittiumcu:0.23625456744496192707):0.09679164194099675045,Spiromyces:0.23974833462196951106):0.03551735670316415877):0.09665633286325017726):0.05211098041147263493,((Basranarum:0.04501951516869184128,Bashaptosp:0.05897344405128001482):0.13072939524583382376,(entomophth:0.52394929175365878571,Conidiobol:0.34322679778606252077):0.09674192742194470673):0.01980241305413410480):0.01893923766699252831,((OlpbornoJD:0.22031883587247191869,Olpbrassi1:0.07482781619285071295):0.15465811958708167162,(glomusintr:0.09959334520165656623,scutellosp:0.08999293617761104647):0.15319943407905151078):0.03633661183083022189):0.01338996527280239546,((piptocepha:0.39414825310675327685,rhopalomyc:0.20421131943699122324):0.12775801083444460193,((endogonepi:0.13206335859596635141,(Umbelopsis:0.07390458187626065822,(Phycomyces:0.07138424790162622990,(Cokeromyce:0.05503024706777164682,Rhizopusor:0.04899904787308095866):0.04514266338221429448):0.06120380450804234485):0.05353464735694027365):0.06429318067973321948,Mortierell:0.26454163936991520467):0.02680131003910026996):0.01337195607299325115):0.03531958931034775984):0.10498097864248212563,(((Saccharomy:0.20936702924097072565,Candidaalb:0.12544883225396533111):0.07510070315372242844,Yarrowiali:0.15583489818794515891):0.12373124890812731147,Neurospora:0.46684470148450329940):0.11862988092454401778):0.06986483516328612087,((Coprinopsi:0.09764214861113630628,Phanerocha:0.08363619451581058573):0.12345787311830992705,Cryptococu:0.43940611279960906099):0.04159373112271627648,Pucciniagr:0.23786188610587083447):0.0;

**Tree 3 (*Olpidium* united with Mucoromycotina in Zygomycota) Initial guide tree**

((OlpbornoJD,Olpbrassi1,Mortierell,endogonepi,Umbelopsis,Phycomyces,Cokeromyce,Rhizopusor),(piptocepha,rhopalomyc,scutellosp,glomusintr,physoderma,coelomomyc,Conidiobol,entomophth,Basranarum,Bashaptosp,Coemansia2,Spiromyces,dimargaris,Furculomyc,Smittiumcu,Candidaalb,Pucciniagr,Coprinopsi,Phanerocha,Cryptococu,Neurospora,Yarrowiali,Saccharomy,Blastoclad,Catenaria1,AllomyRoze,Allommacr2,neocallif2,Phlyctochy,Chytriohya,rhizoclosm,Rhizophlyc,polychytri,entophcon2,Gaertnerio,Spizellomy,rhizophmac,Batrachoch,synchytriu,Cladochytr,Nowakowski,hyaloraphi,Monoblema2,Monoblepha,Gonapodyas,Caeelegans,Drosophila,Homosapien,Dictyostel,Toxoplasma,Cryptospor,Phytophsoj,Cyanidiosi,Populustri));

**Tree 3 (*Olpidium* united with Mucoromycotina in Zygomycota) Constraint**

((Cryptococu:0.44015639652602217557,(Coprinopsi:0.09742911768098312664,Phanerocha:0.08403276102413420601):0.12314114045540627829):0.04189174178069811350,(((((((Dictyostel:0.48230892060964952739,((Cyanidiosi:0.76729340968319714733,Populustri:0.38592500253257888687):0.08757925020653670178,((Cryptospor:0.40518721094535214977,Toxoplasma:0.40035196656309907093):0.42235613307417135820,Phytophsoj:0.47443359695583958713):0.10999253867576280763):0.03998597686540288076):0.06212010711978884631,(Caeelegans:0.22615760106265342388,(Drosophila:0.18700275610308120244,Homosapien:0.14599390572884388129):0.05661977901417028503):0.31206862422179743044):0.15349104782844441575,((Gonapodyas:0.16547565184578036379,(Monoblema2:0.08941352954232636985,Monoblepha:0.05992452835602232741):0.30945735516044142743):0.03182192525219308676,hyaloraphi:0.36058308156259716304):0.13822392640822492305):0.02914978220146372673,(neocallif2:0.25593813906303780614,((polychytri:0.23759545741608040226,(Nowakowski:0.11864873503557379908,Cladochytr:0.26384311926575559903):0.12958487358348311225):0.04167223657335997894,(((rhizophmac:0.21727484492667395455,Batrachoch:0.13369160010994199372):0.08505224256708042441,(Rhizophlyc:0.42219485920322563643,(Gaertnerio:0.06770044500709715418,(entophcon2:0.22357802029233353469,Spizellomy:0.09117823115408443813):0.07801587506516635684):0.04167259300941931338):0.04425383139642609376):0.01878173004384096206,(synchytriu:0.48055671956159784486,(Phlyctochy:0.26615676173227820200,(rhizoclosm:0.10209120409197509904,Chytriohya:0.14320556279015714263):0.13534362156544263445):0.08474989982437801384):0.07443710078044910028):0.02331823192983970006):0.06738388820658075540):0.01386234334101927278):0.02546032710876267730,((coelomomyc:0.18306667574639667873,((Blastoclad:0.10644309530897322347,Catenaria1:0.20664174383290995207):0.09274082795155652592,(AllomyRoze:0.01193242672095373540,Allommacr2:0.00363044390224815609):0.11225428624092827212):0.05786185247610071969):0.14302378716072614018,physoderma:0.30022398217661655506):0.09533361068538247218):0.04497256321225777398,((scutellosp:0.08676815409008760416,glomusintr:0.10258914775281845200):0.18075474570033303601,(((piptocepha:0.40021258883926136285,rhopalomyc:0.20062233070212340258):0.12906634506694822373,((OlpbornoJD:0.21735534177319618121,Olpbrassi1:0.07774642113429716672):0.18915774093614792783,((endogonepi:0.13202013373713566979,((Phycomyces:0.07155503635215500202,(Cokeromyce:0.05513447441936064619,Rhizopusor:0.04892550728457708414):0.04498172643747311988):0.06090215571122944982,Umbelopsis:0.07409343109268398675):0.05360826256149438945):0.06373585756230087940,Mortierell:0.26521799124765788047):0.03703056380851211388):0.00067330078576672875):0.00726367573165059236,((dimargaris:0.37238544683784791101,(((Furculomyc:0.14987762335226650912,Smittiumcu:0.23609504758574090255):0.09658553079879217407,Spiromyces:0.24063044152674895049):0.03567357979973949378,Coemansia2:0.40379021097415096486):0.09669315282784586574):0.05190458125046573667,((Bashaptosp:0.05877133847103372527,Basranarum:0.04527362019507914781):0.13166512902255239625,(entomophth:0.52385771141151893371,Conidiobol:0.34372815901031883445):0.09649235418907320394):0.02039006692760999412):0.02492222106477533575):0.01155320766438654341):0.02769902136880973351):0.10541324586251075990,(Neurospora:0.46709174821616372686,(Yarrowiali:0.15608350450833019529,(Saccharomy:0.20962582113983274645,Candidaalb:0.12551707766412625866):0.07515056042599184416):0.12409257821156233215):0.11891136198610333108):0.07025227374560867599,Pucciniagr:0.23738892873197420252):0.0;

**Tree 4 (*Olpidium* united with Dikarya) Initial guide tree**

((OlpbornoJD,Olpbrassi1,Neurospora,Yarrowiali,Candidaalb,Saccharomy,Coprinopsi,Cryptococu,Phanerocha,Pucciniagr),(Mortierell,endogonepi,Umbelopsis,Phycomyces,Rhizopusor,Cokeromyce,scutellosp,glomusintr,dimargaris,Coemansia2,Spiromyces,Furculomyc,Smittiumcu,Conidiobol,entomophth,Basranarum,Bashaptosp,piptocepha,rhopalomyc,physoderma,coelomomyc,Blastoclad,Catenaria1,AllomyRoze,Allommacr2,neocallif2,Phlyctochy,Chytriohya,rhizoclosm,Rhizophlyc,polychytri,entophcon2,Gaertnerio,Spizellomy,rhizophmac,Batrachoch,synchytriu,Cladochytr,Nowakowski,hyaloraphi,Monoblema2,Monoblepha,Gonapodyas,Caeelegans,Drosophila,Homosapien,Dictyostel,Toxoplasma,Cryptospor,Phytophsoj,Cyanidiosi,Populustri));

**Tree 4 (*Olpidium* united with Dikarya) Constraint**

((((OlpbornoJD:0.21733903803481649519,Olpbrassi1:0.08163151019148613685):0.18549894203382058522,((((dimargaris:0.37401715336171459381,(Coemansia2:0.40802635040808027389,(Spiromyces:0.24224627417816052555,(Furculomyc:0.15075869689411108832,Smittiumcu:0.23757532878810511123):0.09734826145478228665):0.03431425287218119180):0.09757679912709717185):0.05293008177628695521,((Bashaptosp:0.05939422689408085237,Basranarum:0.04532818016795842064):0.13332875311489034154,(entomophth:0.52634828807430644737,Conidiobol:0.34661082618097427410):0.09631419470159502394):0.02000124516432158495):0.02536528389354879287,((scutellosp:0.08856618817259197252,glomusintr:0.10198958440470712317):0.18500472241602980428,((piptocepha:0.39772551161442976086,rhopalomyc:0.20499449631473398714):0.12892823216567136924,(Mortierell:0.26767092458784280673,(((Phycomyces:0.07152970761593178961,(Rhizopusor:0.04934806639533727279,Cokeromyce:0.05524289710925891356):0.04598479273254797717):0.06133126173578242607,Umbelopsis:0.07423361667340792891):0.05429126884475320852,endogonepi:0.13328229951409159559):0.06371460288632939395):0.02836972574947766237):0.01072155545364726799):0.00444544934447735959):0.02190053256683145363,(((((Gonapodyas:0.16704161877763198940,(Monoblema2:0.09019232062862697630,Monoblepha:0.06011895298034338614):0.31176950190747360070):0.03273291808587242696,hyaloraphi:0.36208215275556787649):0.13954016421608006482,((Dictyostel:0.48506565508079568794,((Populustri:0.38863490505277015208,Cyanidiosi:0.77213999834367719188):0.08779626292930936549,((Toxoplasma:0.40310853295442861111,Cryptospor:0.40776865835346576272):0.42578547469366667677,Phytophsoj:0.47705876286746379256):0.11081600259836493771):0.04059657606330500240):0.06245563869847788052,((Drosophila:0.18809252799426232072,Homosapien:0.14700065579558929674):0.05739452157132017562,Caeelegans:0.22690622941934085466):0.31478881418333881648):0.15497971697449794415):0.02907437796823400880,(((polychytri:0.23927186928844154656,(Nowakowski:0.11894426405532090851,Cladochytr:0.26391149795323615157):0.13251861011881088670):0.04120426744312646189,(((Rhizophlyc:0.42539712967585036063,((Spizellomy:0.09161720706785743407,entophcon2:0.22488679766067815047):0.07785338930182027217,Gaertnerio:0.06794269103717254232):0.04176819454646963498):0.04450154088271535135,(rhizophmac:0.21942710588879307809,Batrachoch:0.13403039437453589944):0.08561479030849522553):0.01889519758058010382,((Phlyctochy:0.26709327451495379346,(rhizoclosm:0.10230341568618228631,Chytriohya:0.14471961444407227537):0.13685650066238269629):0.08472167027005196582,synchytriu:0.48345002352545463076):0.07490108835134533238):0.02323621190913862170):0.06679809745834053292,neocallif2:0.25831609364847735177):0.01485408106191321922):0.02720250531022288393,((coelomomyc:0.18506789488892749773,((AllomyRoze:0.01204680867909225205,Allommacr2:0.00359804578948966095):0.11113650512759051803,(Blastoclad:0.10700978002099326147,Catenaria1:0.20831027078026487231):0.09466721690157327285):0.05862044024087512128):0.14456283538040975989,physoderma:0.30224137620538255034):0.09368975753252130334):0.05516040073592955867):0.01250947899356856692):0.10685155794437993515,(Neurospora:0.47047637340560721775,(Yarrowiali:0.15655670666042464934,(Saccharomy:0.21125028418538921438,Candidaalb:0.12614949174594938808):0.07540589581960474574):0.12487536146686517569):0.12173228146328660126):0.07019320739623464978,(Cryptococu:0.44331586704483527672,(Coprinopsi:0.09747902688723356079,Phanerocha:0.08497591468783399771):0.12335136948623182107):0.04138951889999306633,Pucciniagr:0.23730855104448272952):0.0;

**Tree 5 (*Olpidium* sister to all terrestrial fungi) Initial guide tree**

((Pucciniagr,Cryptococu,Coprinopsi,Phanerocha,Neurospora,Yarrowiali,Saccharomy,Candidaalb,scutellosp,glomusintr,Mortierell,endogonepi,Umbelopsis,Phycomyces,Cokeromyce,Rhizopusor,piptocepha,rhopalomyc,Basranarum,Bashaptosp,Conidiobol,entomophth,dimargaris,Coemansia2,Spiromyces,Furculomyc,Smittiumcu),(physoderma,coelomomyc,Olpbrassi1,OlpbornoJD,Blastoclad,Catenaria1,AllomyRoze,Allommacr2,synchytriu,Cladochytr,Nowakowski,Rhizophlyc,Phlyctochy,Chytriohya,rhizoclosm,polychytri,rhizophmac,Batrachoch,Gaertnerio,entophcon2,Spizellomy,neocallif2,hyaloraphi,Gonapodyas,Monoblema2,Monoblepha,Caeelegans,Drosophila,Homosapien,Dictyostel,Cyanidiosi,Populustri,Phytophsoj,Toxoplasma,Cryptospor));

**Tree 5 (*Olpidium* sister to all terrestrial fungi) Constraint**

(((((Candidaalb:0.12619309859400401619,Saccharomy:0.21120166790435496162):0.07540165952627102264,Yarrowiali:0.15649364781048855089):0.12531253276183004775,Neurospora:0.46970952490032152893):0.12175779620205651188,(((((((polychytri:0.23929625504872942265,(Nowakowski:0.11896932632249240802,Cladochytr:0.26405599388038480946):0.13238242170166961142):0.04136669336451716944,(((rhizophmac:0.21938067101130886982,Batrachoch:0.13401303763383215495):0.08569966236535725423,(((Spizellomy:0.09167057820825158521,entophcon2:0.22480457752609270261):0.07779540869454329433,Gaertnerio:0.06799887392862823121):0.04178572228434463548,Rhizophlyc:0.42531745240991125634):0.04447759877554455926):0.01890746814452329283,(synchytriu:0.48342891507826818431,(Phlyctochy:0.26716967876128949744,(Chytriohya:0.14463200593417813167,rhizoclosm:0.10238502901796181499):0.13692847519253140143):0.08482992213863506381):0.07478136162880048843):0.02313379799155632771):0.06712731205424990466,neocallif2:0.25810312059506118620):0.01517902952161482612,((hyaloraphi:0.36207489628020572692,((Monoblepha:0.06007738010218717506,Monoblema2:0.09020807864055220560):0.31182624588005308786,Gonapodyas:0.16715757044673781873):0.03274564256137064977):0.13972244797097396751,((Dictyostel:0.48524127723677251689,((Cyanidiosi:0.77187385327697544124,Populustri:0.38884896888042719265):0.08778448258545908101,(Phytophsoj:0.47727429386221087437,(Toxoplasma:0.40314564534553187780,Cryptospor:0.40779215428942117150):0.42563476325299537395):0.11089770016281479126):0.04053579323876205925):0.06262033406171906014,((Drosophila:0.18808176844701005037,Homosapien:0.14698973823273159267):0.05744607374427809082,Caeelegans:0.22681214055211926239):0.31449025299110877629):0.15485291789640515114):0.02861065218276412012):0.02717210116221297628,((coelomomyc:0.18528335592084591021,((AllomyRoze:0.01204416606137600695,Allommacr2:0.00360029621513239563):0.11122921928596421481,(Blastoclad:0.10708587961862742199,Catenaria1:0.20823142958166160343):0.09461844711219799531):0.05841849139667294050):0.14487245511133850506,physoderma:0.30237766397077064928):0.09366558495758878222):0.05571920679201977644,(Olpbrassi1:0.08175459643644779339,OlpbornoJD:0.21689363497842426010):0.19629740533022421722):0.00000094370619853227,(((scutellosp:0.08856445165160396760,glomusintr:0.10193332341773507499):0.18499107548912810395,((piptocepha:0.39723539383743167175,rhopalomyc:0.20525660347865573585):0.12888435285690771903,((((Phycomyces:0.07163930336657112208,(Cokeromyce:0.05526151655661375867,Rhizopusor:0.04936129973939627769):0.04587023409868799112):0.06132612544486075457,Umbelopsis:0.07421971974013992501):0.05404785487758394236,endogonepi:0.13327567743126675359):0.06352912458123427530,Mortierell:0.26805114531466034400):0.02850330861841373839):0.01062379035411001663):0.00482029890347602727,((dimargaris:0.37438598339062412190,(Coemansia2:0.40800426102885956059,(Spiromyces:0.24211713687894162450,(Furculomyc:0.15065015323390801294,Smittiumcu:0.23769617397683817650):0.09762521136746361849):0.03431731167503008356):0.09766710430467183401):0.05291712172986418744,((entomophth:0.52644996268695121167,Conidiobol:0.34625998897253434938):0.09628566423824655440,(Basranarum:0.04528885074275785377,Bashaptosp:0.05945408538095312695):0.13348568387809445213):0.01969236952655255973):0.02563270804067278516):0.02264158393099513153):0.11680546603128649785):0.06986107267726801573,((Phanerocha:0.08488177446571419649,Coprinopsi:0.09758271908695304031):0.12307627053298829389,Cryptococu:0.44352468713583576010):0.04231907768900119993,Pucciniagr:0.23691045883847511311):0.0;

**Tree 6 (*Olpidium* united with Blastocladiomycota) Initial guide tree**

((OlpbornoJD,Olpbrassi1,physoderma,coelomomyc,Blastoclad,Catenaria1,AllomyRoze,Allommacr2),(Pucciniagr,Cryptococu,Coprinopsi,Phanerocha,Neurospora,Yarrowiali,Saccharomy,Candidaalb,scutellosp,glomusintr,Mortierell,endogonepi,Umbelopsis,Phycomyces,Cokeromyce,Rhizopusor,piptocepha,rhopalomyc,Basranarum,Bashaptosp,Conidiobol,entomophth,dimargaris,Coemansia2,Spiromyces,Furculomyc,Smittiumcu,synchytriu,Cladochytr,Nowakowski,Rhizophlyc,Phlyctochy,Chytriohya,rhizoclosm,polychytri,rhizophmac,Batrachoch,Gaertnerio,entophcon2,Spizellomy,neocallif2,hyaloraphi,Gonapodyas,Monoblema2,Monoblepha,Caeelegans,Drosophila,Homosapien,Dictyostel,Cyanidiosi,Populustri,Phytophsoj,Toxoplasma,Cryptospor));

**Tree 6 (*Olpidium* united with Blastocladiomycota) Constraint**

((((((((((Chytriohya:0.14647848141320521376,rhizoclosm:0.09987039934883157333):0.13527942429597128449,Phlyctochy:0.27044706079735725268):0.08556158791290037480,synchytriu:0.48398043614603247775):0.07296121133484069488,((rhizophmac:0.21709037096119129795,Batrachoch:0.13617896009720970030):0.08769288217246921924,((Nowakowski:0.11610972290400511930,Cladochytr:0.26366866685530554459):0.14244169439423123857,polychytri:0.23838053888662216750):0.04013396175438922742):0.01836480748637364310):0.01074807571590700138,(Rhizophlyc:0.42244139966070992642,((entophcon2:0.23024390952329457094,Spizellomy:0.08884888308590536965):0.08304558673467248020,Gaertnerio:0.06731517788220510445):0.04503343585235213692):0.04554149583902453169):0.07337594622338781958,neocallif2:0.26265948391418786345):0.01707165350970615836,((hyaloraphi:0.36470405906091907244,((Monoblema2:0.08967893913935737205,Monoblepha:0.06064959520457785525):0.31457543687333217042,Gonapodyas:0.16442804110661246586):0.03027280717816736161):0.14150845525147393023,((Dictyostel:0.48516034977865518663,((Phytophsoj:0.47733373620942520787,(Cryptospor:0.40734890625405428821,Toxoplasma:0.40312928074250153898):0.42581188303708650267):0.11071215417628833821,(Cyanidiosi:0.77066109255395565114,Populustri:0.38808828122082933421):0.08812811154818862847):0.03971709478431690449):0.06321911036856094068,(Caeelegans:0.22658802543366596272,(Drosophila:0.18793385738275808494,Homosapien:0.14706833593216053768):0.05755808094239224515):0.31377052992728460268):0.14914178359522906048):0.03170956706946416459):0.04890104132087293165,(((OlpbornoJD:0.21770567894618297888,Olpbrassi1:0.08178707568584926701):0.18555916364807853447,(physoderma:0.30048307291439158018,(((Blastoclad:0.10776634629794604181,Catenaria1:0.20719398901547059211):0.09760449598306890351,(AllomyRoze:0.01219115400146418907,Allommacr2:0.00337391829177308578):0.10714487719677759370):0.05848067134657868038,coelomomyc:0.18482176249848294991):0.14590768469147658015):0.12225006507752263218):0.01425599580904423312,((((entomophth:0.52808901620896653295,Conidiobol:0.34568152387278749949):0.09577783470797548993,(Basranarum:0.04538009247536655549,Bashaptosp:0.05958771537817786346):0.13443906379952602625):0.01809749817581973036,(dimargaris:0.37532562424975568494,(Coemansia2:0.40820831011471581107,(Spiromyces:0.24314267207291717643,(Furculomyc:0.14970448689043178159,Smittiumcu:0.23837213579483867987):0.09674300734929462686):0.03445273780023205140):0.09766549806823472679):0.05268808831386898039):0.02662774721127526140,(((piptocepha:0.39839436033836145290,rhopalomyc:0.20355489398632922726):0.12846357246985767864,(Mortierell:0.26684317573390287093,(endogonepi:0.13429091902582748763,((Phycomyces:0.07130226503222587831,(Cokeromyce:0.05523773441962841202,Rhizopusor:0.04963240239676557780):0.04603914186787153029):0.06110384351475722092,Umbelopsis:0.07457441770444518669):0.05237009094366101847):0.06348362279764660498):0.02833265187230164101):0.01001777952133318939,(scutellosp:0.08897092975158761574,glomusintr:0.10155476511765121339):0.18469249943674309611):0.00583147998645728270):0.02405469905457413918):0.02361255211863800066):0.10424327402413195720,((Yarrowiali:0.15822966204785215982,(Saccharomy:0.21058131353840106592,Candidaalb:0.12649780668953056195):0.07437982674201819300):0.12372659275852228300,Neurospora:0.46915204311442187946):0.11916517182000800623):0.07082435196076772299,((Coprinopsi:0.09660577293271715993,Phanerocha:0.08569182360512771213):0.12392104588901353690,Cryptococu:0.44365988323678001715):0.04294860167050425226,Pucciniagr:0.23859841866958861334):0.0;

**Tree 7 (*Olpidium* sister to all other fungi) Initial guide tree**

((Caeelegans,Drosophila,Homosapien,Dictyostel,Cyanidiosi,Populustri,Phytophsoj,Toxoplasma,Cryptospor,OlpbornoJD,Olpbrassi1),(hyaloraphi,Gonapodyas,Monoblema2,Monoblepha,neocallif2,synchytriu,Cladochytr,Nowakowski,Rhizophlyc,Phlyctochy,Chytriohya,rhizoclosm,polychytri,rhizophmac,Batrachoch,Gaertnerio,entophcon2,Spizellomy,physoderma,coelomomyc,Blastoclad,Catenaria1,AllomyRoze,Allommacr2,Pucciniagr,Cryptococu,Coprinopsi,Phanerocha,Neurospora,Yarrowiali,Saccharomy,Candidaalb,scutellosp,glomusintr,Mortierell,endogonepi,Umbelopsis,Phycomyces,Cokeromyce,Rhizopusor,piptocepha,rhopalomyc,Basranarum,Bashaptosp,Conidiobol,entomophth,dimargaris,Coemansia2,Spiromyces,Furculomyc,Smittiumcu));

**Tree 7 (*Olpidium* sister to all other fungi) Constraint**

((Cryptococu:0.44209711860091660540,(Phanerocha:0.08529570121108501213,Coprinopsi:0.09592933206125472956):0.12215201710583635519):0.04161689608401111795,((((((Caeelegans:0.22375217621279441804,(Homosapien:0.14502543147194191242,Drosophila:0.18804021148734562097):0.05781510901477879272):0.31389801968284197775,(Dictyostel:0.47805164393190607841,((Populustri:0.38678205954072086836,Cyanidiosi:0.76650153059669123845):0.08944521338360218732,(Phytophsoj:0.47310232523967715723,(Toxoplasma:0.40221597148857751725,Cryptospor:0.40312745176864783758):0.42243779571690565078):0.10737600187151456754):0.03841757721588024560):0.06572094809305069341):0.19755821977989690419,(Olpbrassi1:0.08001515766262703999,OlpbornoJD:0.21659684470612761142):0.19303735593320481279):0.00383700090170065351,(((dimargaris:0.37307121510254037666,(Coemansia2:0.40459993169527613821,(Spiromyces:0.23995032661958709541,(Furculomyc:0.15007521003293897155,Smittiumcu:0.23512683109921200808):0.09701338908944248662):0.03430075357447084222):0.09594131781613227106):0.05258725032039870267,((entomophth:0.52203324500698167210,Conidiobol:0.34539505265661751832):0.09427046088845283944,(Basranarum:0.04457891193180026773,Bashaptosp:0.05944292513087365282):0.13407594712028861328):0.01947942326160167353):0.02322486501477931745,((((endogonepi:0.13240506646691796444,(Umbelopsis:0.07387432480413735902,(Phycomyces:0.07121373014487401842,(Cokeromyce:0.05492247169837594090,Rhizopusor:0.04891508974724920189):0.04540007061606476241):0.06080055641602749583):0.05369457494894660371):0.06175427954973063821,Mortierell:0.26573103891415184030):0.02854503457214603232,(piptocepha:0.39324496562395055976,rhopalomyc:0.20327726906216972425):0.12758363318661086749):0.00987514624804921443,(scutellosp:0.08828988261391858272,glomusintr:0.10060076669993289755):0.18424834474973342258):0.00708394822439827564):0.02695377784526421794):0.02020584631318516483,((physoderma:0.29778986997063427999,(coelomomyc:0.18422225779476383845,((AllomyRoze:0.01187988101748536571,Allommacr2:0.00362242472400644373):0.11187961473430037274,(Catenaria1:0.20631176570578982354,Blastoclad:0.10601258318197960551):0.09328937206403717375):0.05713340044498732562):0.14354126665462504930):0.08968781124021821261,(((((Monoblepha:0.06571561320235326520,Monoblema2:0.08329392151939432609):0.30210760175943152106,Gonapodyas:0.15496234245257861639):0.03870771496177664861,hyaloraphi:0.36375137634433424294):0.15298742102680362720,(((((Chytriohya:0.14661084697438761526,rhizoclosm:0.09948944792727994202):0.12810806993826703848,Phlyctochy:0.26786340379180351157):0.09402461029445247087,synchytriu:0.47471457472865469240):0.06990675708930460530,(((rhizophmac:0.21765132158309841159,Batrachoch:0.13048600601529480869):0.08147879386520523459,polychytri:0.26611738915872318323):0.02005994971503965302,(Rhizophlyc:0.41842941387245807805,(Gaertnerio:0.06753053934140797188,(Spizellomy:0.08994712643020415233,entophcon2:0.22465033160595079820):0.08141977315685805516):0.04249070188855975233):0.04208862677192211355):0.01689621206847368232):0.02293597679963603822,(Cladochytr:0.24889136948371332059,Nowakowski:0.11695254617145457721):0.17073911280941814206):0.04776985153860173472):0.01912941502057644363,neocallif2:0.25846711319836401799):0.02635881803887981517):0.04748679092022260739):0.10502104307402669592,(Neurospora:0.46615950699336872187,(Yarrowiali:0.15642744158585858427,(Saccharomy:0.20863175185065480077,Candidaalb:0.12572970989155035637):0.07415521073137959496):0.12373086446169416497):0.11957928848372720299):0.06993691897261299084,Pucciniagr:0.23869734917756255110):0.0;

**Tree 8 (*Olpidium* sister to all terrestrial fungi and Blastocladiomycota) Initial guide tree**

((synchytriu,Cladochytr,Nowakowski,Rhizophlyc,Phlyctochy,Chytriohya,rhizoclosm,polychytri,rhizophmac,Batrachoch,Gaertnerio,entophcon2,Spizellomy,neocallif2,hyaloraphi,Gonapodyas,Monoblema2,Monoblepha,Caeelegans,Drosophila,Homosapien,Dictyostel,Cyanidiosi,Populustri,Phytophsoj,Toxoplasma,Cryptospor,OlpbornoJD,Olpbrassi1),(physoderma,coelomomyc,Blastoclad,Catenaria1,AllomyRoze,Allommacr2,Pucciniagr,Cryptococu,Coprinopsi,Phanerocha,Neurospora,Yarrowiali,Saccharomy,Candidaalb,scutellosp,glomusintr,Mortierell,endogonepi,Umbelopsis,Phycomyces,Cokeromyce,Rhizopusor,piptocepha,rhopalomyc,Basranarum,Bashaptosp,Conidiobol,entomophth,dimargaris,Coemansia2,Spiromyces,Furculomyc,Smittiumcu));

**Tree 8 (*Olpidium* sister to all terrestrial fungi and Blastocladiomycota) Constraint**

(((Neurospora:0.46921876325445827094,(Yarrowiali:0.15751700275591992706,(Saccharomy:0.21142909941787957351,Candidaalb:0.12656955586315468687):0.07513853370399745102):0.12626140583811543139):0.12175189316080767266,(((coelomomyc:0.18243546410895644660,((AllomyRoze:0.01212692555721769580,Allommacr2:0.00343806574878666215):0.11028228946582903369,(Blastoclad:0.10892027190278971704,Catenaria1:0.20692622620510073284):0.09594327863654183997):0.05840507711308978722):0.14086451212388906296,physoderma:0.30278524263972078634):0.11808240614716121464,(((((hyaloraphi:0.36418731727650743846,(Gonapodyas:0.16701981390939915628,(Monoblema2:0.08995159283917839033,Monoblepha:0.06043427297317498226):0.31423495095940412458):0.03135245252506459190):0.14387057272063130675,((Caeelegans:0.22625164669615766000,(Drosophila:0.18841241671569308735,Homosapien:0.14742858054959434577):0.05831994313328531243):0.31510912870823148957,(Dictyostel:0.48576010028269800411,((Cyanidiosi:0.77311238629170253578,Populustri:0.38960200161865837787):0.08789742697280747419,(Phytophsoj:0.47808026041317319699,(Toxoplasma:0.40471684602531826647,Cryptospor:0.40786272538124318521):0.42750405234571131619):0.11069110162447744206):0.04143724420772576522):0.06357091705417217131):0.15031949777295003368):0.03256597663785816554,(((polychytri:0.23732473207585061403,(Nowakowski:0.11886824214812712064,Cladochytr:0.25985437581002462926):0.14335503633707649684):0.04273144139184923557,((synchytriu:0.48436899252459841847,(Phlyctochy:0.26838701013947968699,(Chytriohya:0.14646222409601403203,rhizoclosm:0.10048105876193316743):0.13490876021056455447):0.08772101499558272730):0.07483231385077901421,((rhizophmac:0.22066409781815543578,Batrachoch:0.13361216973888828674):0.08651668900058680223,(((Spizellomy:0.09126235382001347818,entophcon2:0.22613644968829385573):0.07677633570626475612,Gaertnerio:0.06851910358642568366):0.04382477846051374692,Rhizophlyc:0.42610526005484322676):0.04460565578739894566):0.01677410396549497765):0.02024813864632116905):0.06919532143366687038,neocallif2:0.25920089078603497645):0.01675030944964662344):0.06174717297203079214,(Olpbrassi1:0.08124440138444857795,OlpbornoJD:0.21802962870167300058):0.20355853064211368308):0.00000094370619853227,(((dimargaris:0.37538399604579286706,(Coemansia2:0.40923517140139237247,(Spiromyces:0.24311548715087946682,(Furculomyc:0.15065050288106832110,Smittiumcu:0.23865919424826992357):0.09723498751925599426):0.03437524215927451054):0.09733384605848220683):0.05270426350698414336,((entomophth:0.52883962427638131487,Conidiobol:0.34742770605369616144):0.09618581566574348207,(Basranarum:0.04554623297306897856,Bashaptosp:0.05956977787108978795):0.13409411796056386335):0.01919936676729779371):0.02680318119229719090,(((piptocepha:0.39728837447468923205,rhopalomyc:0.20491496551090390699):0.12940931834654528787,(Mortierell:0.26804835119413744637,(endogonepi:0.13400959510730070523,((Phycomyces:0.07158279586000125538,(Rhizopusor:0.04961691898381572624,Cokeromyce:0.05533610733737343718):0.04610409797179325364):0.06137264491655641113,Umbelopsis:0.07447159884337746505):0.05338694584933691145):0.06373603876136381408):0.02815163386203656942):0.01032740078933475948,(scutellosp:0.08848016256384463873,glomusintr:0.10219715334639448145):0.18466606449895975328):0.00547616064840244100):0.02737833702051714613):0.02663223432067301361):0.09744743883070323964):0.06749800549254926507,((Coprinopsi:0.09747593164924331366,Phanerocha:0.08544004416909445343):0.12268039620440843174,Cryptococu:0.44561194171072721071):0.04384629999214703644,Pucciniagr:0.23788624658814425117):0.0;

**Tree 9 (*Olpidium* united with Neocallimastigomycota) Initial guide tree**

((neocallif2,OlpbornoJD,Olpbrassi1),(hyaloraphi,Gonapodyas,Monoblema2,Monoblepha,Caeelegans,Drosophila,Homosapien,Dictyostel,Cyanidiosi,Populustri,Phytophsoj,Toxoplasma,Cryptospor,synchytriu,Cladochytr,Nowakowski,Rhizophlyc,Phlyctochy,Chytriohya,rhizoclosm,polychytri,rhizophmac,Batrachoch,Gaertnerio,entophcon2,Spizellomy,physoderma,coelomomyc,Blastoclad,Catenaria1,AllomyRoze,Allommacr2,Pucciniagr,Cryptococu,Coprinopsi,Phanerocha,Neurospora,Yarrowiali,Saccharomy,Candidaalb,scutellosp,glomusintr,Mortierell,endogonepi,Umbelopsis,Phycomyces,Cokeromyce,Rhizopusor,piptocepha,rhopalomyc,Basranarum,Bashaptosp,Conidiobol,entomophth,dimargaris,Coemansia2,Spiromyces,Furculomyc,Smittiumcu));

**Tree 9 (*Olpidium* united with Neocallimastigomycota) Constraint**

(((((((((AllomyRoze:0.01186860345874615232,Allommacr2:0.00360213277102987067):0.10992822184031092458,(Blastoclad:0.10464338753346008604,Catenaria1:0.20606848461374430159):0.09277306766705706309):0.05350114692889307300,coelomomyc:0.18856493779085253815):0.14114263494932946519,physoderma:0.30110011457186203199):0.08798573944009890901,((hyaloraphi:0.35995124840868891747,((Monoblema2:0.08536793918096631517,Monoblepha:0.06332146825530038381):0.30055732796632073089,Gonapodyas:0.15462037381530965852):0.03731426410357614742):0.14215970293271540381,(((Nowakowski:0.11815102106338179477,Cladochytr:0.25146402800946576761):0.11401106909052288740,synchytriu:0.48225606771607493206):0.05119077350541131194,((polychytri:0.26418189444369927221,(((Spizellomy:0.08916605342125021039,entophcon2:0.22452747517167737090):0.07111113692169031275,Gaertnerio:0.06554814247614666745):0.06921256798016725553,(Batrachoch:0.13151559652413649659,rhizophmac:0.21769825769061412757):0.08109021299587129694):0.02182757226862648212):0.01954014901070000310,(Rhizophlyc:0.39459288121811814110,(Phlyctochy:0.26687872287244118752,(Chytriohya:0.14906912505494276933,rhizoclosm:0.10159956970023134581):0.10299921721519433859):0.13584570540545459760):0.04792977113094835234):0.02758907129763143312):0.04828940187749276858):0.02888489261417373499):0.02824329340369301222,((Dictyostel:0.47606305927480008222,((Cyanidiosi:0.75834244543568440911,Populustri:0.38524573972159759094):0.08988354731522145424,((Toxoplasma:0.39660427503061312926,Cryptospor:0.40151105601187264682):0.41790262339070050057,Phytophsoj:0.46905997407270405564):0.10591288880371045600):0.04031778640617547649):0.06414141905477566130,((Drosophila:0.18600965994934581804,Homosapien:0.14418708395178819748):0.05695976253797009614,Caeelegans:0.22326343445924839992):0.30842368869132308351):0.16584599887024661924):0.03596784803979456713,((neocallif2:0.31135218591156527124,(Olpbrassi1:0.07809682947544051290,OlpbornoJD:0.21676829023220453574):0.19223027864743771898):0.00139037209068525180,(((dimargaris:0.37155874684598172442,(Coemansia2:0.40240884100402890544,(Spiromyces:0.24050034592403848888,(Furculomyc:0.14878718203377033769,Smittiumcu:0.23340872178030008799):0.09484264375454788676):0.03330919338305592114):0.09412274101753650979):0.05213586947471270799,((Conidiobol:0.34381252575068138588,entomophth:0.51966116825162056436):0.09338191780138369036,(Basranarum:0.04421575628970129263,Bashaptosp:0.05922330234591423326):0.13316058020549026431):0.01823776490511670387):0.02464118188340776172,((scutellosp:0.08788154496230198010,glomusintr:0.10018656979430672072):0.18531139151092096418,((Mortierell:0.26318699603670892007,((Umbelopsis:0.07307555086424066815,(Phycomyces:0.07059860764174383241,(Cokeromyce:0.05411626613213075393,Rhizopusor:0.04883192278262458302):0.04543825566684273065):0.06078925885940415774):0.05239621075284234264,endogonepi:0.13164386318696966183):0.06220101012063708468):0.02832714247877552924,(piptocepha:0.39626228619726683311,rhopalomyc:0.19929462475305512181):0.12781123101376778983):0.00942421481312203865):0.00673249455195079789):0.02393246341379971776):0.02704502746972950455):0.10131155041055608934,(Neurospora:0.46283771882451651969,(Yarrowiali:0.15565553339963805746,(Candidaalb:0.12512065535340136102,Saccharomy:0.20674552565975523599):0.07334952029078359304):0.12156368418944189791):0.11905315774462056633):0.06879968165299403526,((Phanerocha:0.08510448755543047195,Coprinopsi:0.09477287540799174403):0.12352574002417977816,Cryptococu:0.43643637007022567653):0.04173385291006291575,Pucciniagr:0.23529309639424988987):0.0;

**Tree 10 (*Olpidium* united with Monoblepharidomycetes) Initial guide tree**

((OlpbornoJD,Olpbrassi1,hyaloraphi,Gonapodyas,Monoblema2,Monoblepha),(Caeelegans,Drosophila,Homosapien,Dictyostel,Cyanidiosi,Populustri,Phytophsoj,Toxoplasma,Cryptospor,neocallif2,synchytriu,Cladochytr,Nowakowski,Rhizophlyc,Phlyctochy,Chytriohya,rhizoclosm,polychytri,rhizophmac,Batrachoch,Gaertnerio,entophcon2,Spizellomy,physoderma,coelomomyc,Blastoclad,Catenaria1,AllomyRoze,Allommacr2,Pucciniagr,Cryptococu,Coprinopsi,Phanerocha,Neurospora,Yarrowiali,Saccharomy,Candidaalb,scutellosp,glomusintr,Mortierell,endogonepi,Umbelopsis,Phycomyces,Cokeromyce,Rhizopusor,piptocepha,rhopalomyc,Basranarum,Bashaptosp,Conidiobol,entomophth,dimargaris,Coemansia2,Spiromyces,Furculomyc,Smittiumcu));

**Tree 10 (*Olpidium* united with Monoblepharidomycetes) Constraint**

(((((((dimargaris:0.37319645692343367260,(Coemansia2:0.40743217508008672256,((Furculomyc:0.15047623789618305712,Smittiumcu:0.23809162172756836995):0.09694571445667549137,Spiromyces:0.24278274223604209836):0.03377335455756022564):0.09820808941103055523):0.05265612302227641595,((Basranarum:0.04535895497856121117,Bashaptosp:0.05948555804617550180):0.13350411325963895681,(entomophth:0.52630246483312137507,Conidiobol:0.34688753233686314692):0.09625408265933148300):0.01919367981608996465):0.02423503576277563978,(((piptocepha:0.39565286301106056266,rhopalomyc:0.20587511802232938418):0.12967207760464494193,(Mortierell:0.26617194873204419459,(((Phycomyces:0.07127184506303740896,(Cokeromyce:0.05514597398040395337,Rhizopusor:0.04941704558587809576):0.04615425554557574944):0.06072163547485368712,Umbelopsis:0.07440324490895101894):0.05292301927700599623,endogonepi:0.13438451727160768967):0.06455906451790840994):0.02628217298119786202):0.01060209208401783308,(scutellosp:0.08821637304176423167,glomusintr:0.10183267180959543197):0.18485801566841267296):0.00731735790510188670):0.03318436612487751791,((neocallif2:0.25862705714820671821,((polychytri:0.23752453935830386555,(Nowakowski:0.12364178337665006346,Cladochytr:0.25931893516462339866):0.13787812173230412638):0.04251498137793347648,((synchytriu:0.48365706289348336933,(Phlyctochy:0.26622403462233273919,(rhizoclosm:0.09979128221150156131,Chytriohya:0.14674034628342183528):0.13813873646085800262):0.08248165159547718450):0.07502660264676253021,((rhizophmac:0.21951393510735467407,Batrachoch:0.13404842352345086587):0.08384157537544921490,(Rhizophlyc:0.42640829028438126391,(Gaertnerio:0.06643912999436546452,(Spizellomy:0.09194039518914186304,entophcon2:0.22440219507498129770):0.07820903937172567211):0.04016888648299042630):0.04537362639886578786):0.02055154078031408080):0.01940370845775458986):0.06764847522074197983):0.02800839340327727217,(((Caeelegans:0.22706726381987568653,(Drosophila:0.18966790213290821154,Homosapien:0.14583709360082153239):0.05717029989767143106):0.31527261189450983547,(Dictyostel:0.48678618542812918113,((Populustri:0.39138744017575949341,Cyanidiosi:0.77282423987059234438):0.08869395333316440477,(Phytophsoj:0.47569787144117997579,(Toxoplasma:0.40430094934101523663,Cryptospor:0.40742008773009813494):0.42590166620389291463):0.10987345432359162112):0.03814677199125198620):0.06198522714902349184):0.16257494717505224791,(physoderma:0.30710907908467355298,(coelomomyc:0.18885737624836743120,((AllomyRoze:0.01201439161818331977,Allommacr2:0.00369359177022702580):0.11385841934098578365,(Blastoclad:0.10654636905655655299,Catenaria1:0.20845863913479095730):0.09255957326299753263):0.05599230431308051259):0.14367952928361046872):0.07701148706541101341):0.03381877430356449582):0.04740998738311674038):0.01104885495251208515,((OlpbornoJD:0.21573764059302336094,Olpbrassi1:0.08400225926245333263):0.19575185679271114858,((Gonapodyas:0.17077028367377594664,(Monoblema2:0.08446579452612498762,Monoblepha:0.06404267693014871476):0.30207348458175148354):0.03487030230373757561,hyaloraphi:0.36386240053895363422):0.20188723748044226647):0.00263198955592958920):0.10703448338980121879,(((Saccharomy:0.21115060246653849507,Candidaalb:0.12607368888803105467):0.07414238745154860810,Yarrowiali:0.15833548392345700440):0.12268554872785436549,Neurospora:0.47299663472087832838):0.12166673808508912669):0.06942919034758054941,(Cryptococu:0.44622652015237967982,(Coprinopsi:0.09649777503399972756,Phanerocha:0.08608873210835595036):0.12099252791043299537):0.04322361191027781352,Pucciniagr:0.23892780391926649042):0.0;

**Tree 11 (*Olpidium* united with "Core chytrid clade") Initial guide tree**

((OlpbornoJD,Olpbrassi1,synchytriu,Cladochytr,Nowakowski,Rhizophlyc,Phlyctochy,Chytriohya,rhizoclosm,polychytri,rhizophmac,Batrachoch,Gaertnerio,entophcon2,Spizellomy),(neocallif2,hyaloraphi,Gonapodyas,Monoblema2,Monoblepha,Caeelegans,Drosophila,Homosapien,Dictyostel,Cyanidiosi,Populustri,Phytophsoj,Toxoplasma,Cryptospor,physoderma,coelomomyc,Blastoclad,Catenaria1,AllomyRoze,Allommacr2,Pucciniagr,Cryptococu,Coprinopsi,Phanerocha,Neurospora,Yarrowiali,Saccharomy,Candidaalb,scutellosp,glomusintr,Mortierell,endogonepi,Umbelopsis,Phycomyces,Cokeromyce,Rhizopusor,piptocepha,rhopalomyc,Basranarum,Bashaptosp,Conidiobol,entomophth,dimargaris,Coemansia2,Spiromyces,Furculomyc,Smittiumcu));

**Tree 11 (*Olpidium* united with "Core chytrid clade") Constraint**

(((Neurospora:0.46853544065186819578,(Yarrowiali:0.15723400139199994463,(Saccharomy:0.20973613237259761122,Candidaalb:0.12578139826793349743):0.07465428870094603653):0.12309233668927761773):0.12103647753401791665,(((((Coemansia2:0.40730707130648402536,((Furculomyc:0.14971741138255093007,Smittiumcu:0.23695155339594983768):0.09614632871369187261,Spiromyces:0.24217586494237366979):0.03437852487047003691):0.09853453237464737668,dimargaris:0.37283871725241068962):0.05038095854266971124,((Conidiobol:0.34550334637187291031,entomophth:0.52632988781727585437):0.09573332690793801003,(Basranarum:0.04493853645059715796,Bashaptosp:0.05945319641016726109):0.13503370017951965565):0.01908237437796600489):0.02510042393302952535,((glomusintr:0.10104034500842044852,scutellosp:0.08847618999174157450):0.18511976715344533040,((piptocepha:0.39498520577409612642,rhopalomyc:0.20469804741990504926):0.12890230278546954690,(Mortierell:0.26681140566007743864,(endogonepi:0.13474942367776779339,(Umbelopsis:0.07438953416482060876,((Cokeromyce:0.05496452259190559575,Rhizopusor:0.04922134785570358995):0.04558566091579067947,Phycomyces:0.07157606250922049496):0.06089065283943092405):0.05217291784424454848):0.06345468196780475190):0.02525119481769336552):0.00923812572583780980):0.00874249692773185032):0.03214500913633789453,((((physoderma:0.30834727138740120056,(coelomomyc:0.18309736848163835066,((AllomyRoze:0.01190622986397330517,Allommacr2:0.00364181326313661085):0.11034160419661502572,(Blastoclad:0.10620101923884449346,Catenaria1:0.20786052141390035097):0.09550661531476890842):0.06078571122070967198):0.13841477540315719197):0.08965483573066831968,((hyaloraphi:0.36182823451983464613,((Monoblema2:0.08826393740258549336,Monoblepha:0.06121515771506232800):0.30537256393252232289,Gonapodyas:0.16948845084922678494):0.03641428694167652552):0.13809718773898232991,((Caeelegans:0.22570784012806793806,(Drosophila:0.18729987820784307906,Homosapien:0.14652279225382128236):0.05711818011734785122):0.31213393438333991714,(Dictyostel:0.48217609922403192879,((Cyanidiosi:0.76907628108104231224,Populustri:0.39001539151556147145):0.08773231853823157400,(Phytophsoj:0.47586210467209388852,(Toxoplasma:0.40260927144314900827,Cryptospor:0.40449939985251048835):0.42327128191484780784):0.10958047781241887775):0.04004705665164366712):0.06319536948132420395):0.15242795131232186878):0.02221178321628540855):0.02554856916024702412,neocallif2:0.26682344220904313792):0.02635403984959600390,((((Gaertnerio:0.06823017249432629383,(entophcon2:0.22613862665398382079,Spizellomy:0.09090869258968500843):0.07619020011873134379):0.04764234245858785244,Rhizophlyc:0.41881970765110260180):0.04459745589296601476,(((rhizophmac:0.21684397043823552820,Batrachoch:0.13468241528951757635):0.08492475531426209312,((Nowakowski:0.11366288619732427267,Cladochytr:0.26278568258624512843):0.14254480343160805256,polychytri:0.23717268521841358697):0.04143281791834632632):0.01879131586209981802,(synchytriu:0.48044415572800358571,((Chytriohya:0.14655477469855898720,rhizoclosm:0.09784613268879656378):0.13462300603661703224,Phlyctochy:0.26680862590426762671):0.08559383570781546602):0.07227618471461599858):0.01050449582767399963):0.10172502468876508386,(Olpbrassi1:0.07913663256047467898,OlpbornoJD:0.21435856402452449498):0.22308676118384598852):0.00712709333661721081):0.03122909471823933497):0.10900318957643989615):0.07005715602881229520,(Cryptococu:0.44308242546597920164,(Coprinopsi:0.09704006232698311751,Phanerocha:0.08480101462688664771):0.12288449000845746983):0.04262863580549662579,Pucciniagr:0.23821670644479020496):0.0;
